# Water-Dispersible Silica-Polyelectrolyte Nanocomposites Prepared via Acid-Triggered Polycondensation of Silicic Acid and Directed by Polycations

**DOI:** 10.3390/polym8030096

**Published:** 2016-03-22

**Authors:** Philip Overton, Elena Danilovtseva, Erno Karjalainen, Mikko Karesoja, Vadim Annenkov, Heikki Tenhu, Vladimir Aseyev

**Affiliations:** 1 Laboratory of Polymer Chemistry, Department of Chemistry, University of Helsinki, A.I. Virtasen aukio 1, P.O. Box 55, FIN-00014 HY Helsinki, Finland; Philip.Overton@cea.fr (P.O.); Erno.Karjalainen@nottingham.ac.uk (E.K.); Mikko.Karesoja@kemira.com (M.K.); Heikki.Tenhu@helsinki.fi (H.T.); 2 Limnological Institute Siberian Branch of the Russian Academy of Sciences, 3, Ulan-Bator Str., Irkutsk 664033, Russia; danilovtseva@yahoo.com (E.D.); annenkov@lin.irk.ru (V.A.)

**Keywords:** silica, nanoparticles, polyelectrolyte, polycations, condensation, colloid, sodium metasilicate, PDMAEMA, PMOTAI, OEGMA, POEGMA

## Abstract

The present work describes the acid-triggered condensation of silicic acid, Si(OH)_4_, as directed by selected polycations in aqueous solution in the pH range of 6.5–8.0 at room temperature, without the use of additional solvents or surfactants. This process results in the formation of silica-polyelectrolyte (S-PE) nanocomposites in the form of precipitate or water-dispersible particles. The mean hydrodynamic diameter (*d*_h_) of size distributions of the prepared water-dispersible S-PE composites is presented as a function of the solution pH at which the composite formation was achieved. Poly(2-(dimethylamino)ethyl methacrylate) (PDMAEMA) and block copolymers of DMAEMA and oligo(ethylene glycol) methyl ether methacrylate (OEGMA) were used as weak polyelectrolytes in S-PE composite formation. The activity of the strong polyelectrolytes poly(methacryloxyethyl trimethylammonium iodide) (PMOTAI) and PMOTAI-*b*-POEGMA in S-PE formation is also examined. The effect of polyelectrolyte strength and the OEGMA block on the formation of the S-PE composites is assessed with respect to the S-PE composites prepared using the PDMAEMA homopolymer. In the presence of the PDMAEMA_60_ homopolymer (*M*_w_ = 9400 g/mol), the size of the dispersible S-PE composites increases with solution pH in the range pH 6.6–8.1, from *d*_h_ = 30 nm to *d*_h_ = 800 nm. S-PDMAEMA_60_ prepared at pH 7.8 contained 66% silica by mass (TGA). The increase in dispersible S-PE particle size is diminished when directed by PDMAEMA_300_ (*M*_w_ = 47,000 g/mol), reaching a maximum of *d*_h_ = 75 nm. S-PE composites formed using PDMAEMA-*b*-POEGMA remain in the range *d*_h_ = 20–30 nm across this same pH regime. Precipitated S-PE composites were obtained as spheres of up to 200 nm in diameter (SEM) and up to 65% mass content of silica (TGA). The conditions of pH for the preparation of dispersible and precipitate S-PE nanocomposites, as directed by the five selected polyelectrolytes PDMAEMA_60_, PDMAEMA_300_, PMOTAI_60_, PDMAEMA_60_-*b*-POEGMA_38_ and PMOTAI_60_-*b*-POEGMA_38_ is summarized.

## 1. Introduction

Inspiration for this work is derived from the discovery of specialized macromolecules in biological systems which direct the formation of ordered silica structures in the micro- and nanometer scale [1,2,3,4,5,6,7]. Silica nanoparticles are useful as support structures for novel polymer grafts [8,9,10,11] and catalysts [12,13,14], as components in controlled release systems for biologically active molecules [15,16] and as fillers in durable polymeric materials [17]. Deposition of silicates onto polymeric supports has been reported as demonstration of the reverse process [18,19]. The existence of proteins able to direct the condensation of silicates under mild conditions has been revealed in recent studies of enzyme-controlled systems [6,20]. Significantly, these biologically active macromolecules have been found to contain amines and other cationic functional groups. Silicic acid, Si(OH)_4_, has been identified as the dominant silicon-containing compound available to biological systems involved in the controlled synthesis of silica nanostructures [1,21]. The acid catalyzed hydrolysis of sodium silicate (Na_2_SiO_3_, SS) has been utilized for the *in situ* formation of silicic acid (SA) under mild conditions [22,23,24,25]. In the present work, the electrostatic interactions of monomeric and oligomeric SA (generated *in situ* from SS) with synthetic polycations is exploited as means for the preparation silica-polyelectrolyte (S-PE) nanocomposites. These are selectively obtained as either water-stable (dispersible) or precipitated particles from aqueous solutions of the selected precursors.

Chemical syntheses of silica-based materials such as resins and micro-sieves have traditionally required extremes of temperature and pressure. Calcination, thermo-spinning techniques and pyrolysis of precursors are common approaches in this field [26]. Silica–polymer nanocomposites have been successfully prepared by sol–gel synthesis from tetraethyl orthosilicate (Si(OEt)_4_; TEOS) followed by carbonization [27,28,29]. Attempts have been made to optimize this technique [30,31]. However, the potential denaturing activity of alcohols on enzymes has limited the use of silicon alkoxides such as TEOS and tetramethyl orthosilicate (Si(OMe)_4_; TMOS) as precursors of SA [32] because their hydrolysis produces alcohols. Evaporation of alcohol by-products of silicon alkoxide hydrolysis under vacuum, prior to the addition of the desired enzyme to the reaction mixture, has been shown to improve the enzyme activity of the resultant composite [33]. However, enzymatic action of proteins known as “silacateins” [6] have been applied *in vitro* to chemically and spatially direct the preparation of silica by polymerization of TEOS under mild conditions in water at neutral pH [20]. Considering the above, the complete absence of alcohol from the synthetic strategy would benefit these sensitive biomimetic studies. The use of SS as a precursor provides an alcohol-free route to the *in situ* formation of SA. In this case, the two-step sol–gel process often involves the preparation of a silicate solution at low pH and subsequent gelation at neutral pH in the presence of biomolecules and a phosphate buffer. In the work reported here, silicic acid (Si(OH)_4_, SA) is generated *in situ* by acid hydrolysis of sodium silicate (Na_2_SiO_3_, SS).

The majority of dissolved silicon in marine environments is found in the form of silicic acid [1,21,34,35]. Diatom algae, a major subset of phytoplankton, are micro-organisms which utilize dissolved SA in the biogenesis of cellular structures called “frustules.” The silica nanostructures observed in diatoms are both species-specific and hereditary: evidently the biosynthesis of such structures is genetically controlled by functional proteins. Certain genes, first discovered in the diatom *Cylindrotheca fusiformis*, have been suggested as silicon transporters (SITs) [36,37].

Long-chain polyamines (LCPAs) have been shown to be key components in the biological transport of silicon [3,4]. LCPAs are consistently found in different diatoms and marine sponges which are able to form ordered, micro-scale structures through controlled condensation of silicates. Cationic proteins called “silaffins” contain covalently modified lysine units and have been observed to direct the biosilification processes of diatom algae, wherein ordered nanostructures containing silicon are created under biological conditions [3,4]. Consequently, silaffins have been identified as promising components for use in the development of biocompatible, composite materials [38,39,40,41]. Natural silaffins exist in diatom cell walls in tiny amounts and are tedious to extract, leading to a growing interest in synthetic polymers able to perform a similar function. Thus poly(allyl amine) [42] and poly(vinyl amine) [43] have been suggested as such synthetic analogs of natural polyamines. The proximity of the amine groups to the non-polar primary chain of these synthetic polymers weakens their electrostatic interactions with silicates in solution. The tertiary amine in the repeating unit of PDMAEMA is bound to the polymer primary chain by an ester linker and is an effort, in the present work, to mimic the chemistry of silaffins in a more appropriate manner.

In this study, “silica” refers to (SiO_2_)*_n_*, which is prepared from sodium silicate (SS). The acid-catalyzed condensation of silicic acid from solution yields poly(silicic acid) (PSA), a material acknowledged to consist of (SiO_2_)*_n_* with a surface bearing silanol (Si–OH) groups. The PSA which has become physically bound to the polyelectrolytes (PE) is abbreviated to “S.” When the selected polyelectrolyte is bound to silica, the resultant composite is termed S-PE, wherein “-” refers to the interaction of the two species.

Colloidal dispersions of S-PE particles have been observed by dynamic light scattering (DLS) to be stable with respect to size and number over a period of days. Similarly, S-PE precipitates were prepared under the same pH regime using higher concentrations of the SS precursor relative to a constant (10 mM) concentration of amine repeating units. In cases where precipitation was induced by the presence of polymer, the resultant S-PE composite was studied by thermogravimetric analysis (TGA) and scanning electron microscopy (SEM). Selected water-stable S-PE particles were isolated by ultrafiltration and analyzed in the same way.

PDMAEMA is a thermally responsive polymer at high pH. The effect of temperature on the formation of the S-PE composites is beyond the scope of this study.

## 2. Materials and Methods

### 2.1. Materials

2,2′-Azobis(2-methylpropionitrile) (AIBN) (Sigma-Aldrich, St. Louis, MO, USA, 98%) was recrystallized from methanol prior to use. 4-cyanopentanoic acid dithiobenzoate (CPA) (Aldrich, Sigma-Aldrich >97%) was used as received. 2-(dimethylamino)ethyl methacrylate (DMAEMA) (Acros Organics, part of Thermo Fisher Scientific, Bridgewater, NJ, USA, 99%) and poly(ethylene glycol)methyl ether methacrylate (OEGMA) (Aldrich, Average *M*_n_ = 475 g/mol, 99%) were each passed through a 10 cm (70 cm^3^) column of Al_2_O_3_ and filtered before use. DMAEMA was additionally distilled under reduced pressure. The number-average molecular weight (*M*_n_) of the OEGMA oligomers (423 g/mol; 7 ethylene glycol repeating units per oligomer) was calculated by ^1^H nuclear magnetic resonance (NMR) in order to make the necessary stoichiometric calculations. Acetone (VWR, Radnor, PA, USA, HPLC-grade), acetonitrile (VWR, HPLC-grade), CuCl (Aldrich, 99.995%), CuCl_2_ (Aldrich, 99.999%), deuterium oxide (Eurisotop, Saint-Aubin, France), ethyl α-bromoisobutyrate (EBiB) (Aldrich, 98%), 0.10 M NaOH-solution and 0.10 M HCl-solution (FF-chemical, Haukipudas, Finland), *n*-hexane (Aldrich, HPLC-grade), sodium metasilicate pentahydrate (Fluka, a part of Sigma-Aldrich, assay ≥97.0%) tetra-*n*-butylammonium bromide (TBAB) (Aldrich, 99%) and tetrahydrofuran (THF) (Aldrich, HPLC-grade) were used as received. Toluene (Fisher Chemical, Pittsburgh, PA, USA, HPLC-grade) was distilled over metallic sodium prior to use.

### 2.2. Polymer Synthesis and Preparation of Composites

#### 2.2.1. Synthesis of Polymers

PDMAEMA and the related polyelectrolytes used in the present work were synthesized by reversible addition fragmentation chain-transfer (RAFT) [44,45] polymerization and atom-transfer radical polymerization (ATRP) [46]. Full details of all the polymerizations and the characterization of the products can be found in the Supplementary Materials (SM; Figures S1–S8, Tables S1–S3).

As an example, the synthesis of PDMAEMA_60_ via RAFT polymerization was conducted as follows. AIBN (0.021 g, 0.128 mmol), CPA (0.358 g, 1.281 mmol) and DMAEMA (10.011 g, 63.679 mmol) were dissolved in distilled toluene (3 mL) and placed in a 25 mL round bottom flask with a magnetic stirrer bar. The reaction mixture was degassed with three freeze-pump-thaw cycles and placed in a 70 °C oil bath to initiate the reaction. The flask was heated at 70 °C with stirring (19 h) and the reaction was finally quenched by exposing the reaction mixture to air and submerging the flask in liquid nitrogen. At this point, a conversion sample for NMR analysis was taken. The product was precipitated from cold hexane and a second time from acetone. The second precipitate was dissolved in the minimum amount of acetonitrile and concentrated by rotary evaporation. The resulting solution was diluted with water (40 mL) and dialyzed against water (4 L, 3 days, water changed 4 times). The coral-colored product was isolated by freeze-drying (69.7% conversion by ^1^H NMR, 76.9% yield by mass, *M*_n_ = 9400 g/mol by ^1^H NMR end group analysis, *M*_w_ = 9200 g/mol by SEC, *M*_w_/*M*_n_ = 1.12 by SEC). Number of repeating units (^1^H NMR) = 60. ^1^H NMR (500 MHz, D_2_O, protons marked “a”–“e” in SM, Figure S4), chemical shift (δ)/ppm: (–CH_2_–) H^a^ = 1.85; (R–CH_3_) H^b^ = 0.86, 1.06; (–COOCH_2_) H^c^ = 4.10; (–CH_2_–NMe_2_) H^d^ = 2.69; (RN(CH_3_)_2_) H^e^ = 2.27.

Chain extensions of PDMAEMA_60_ to yield PDMAEMA_60_-*b*-POEGMA_38_ were conducted in the same manner as the synthesis of the homopolymer. PDMAEMA fulfilled the role of the macro-RAFT chain transfer agent (SM, Figure S2). The masses and mole ratios of the compounds used in the polymerizations are summarized below (Table 1).

**Figure 1:**
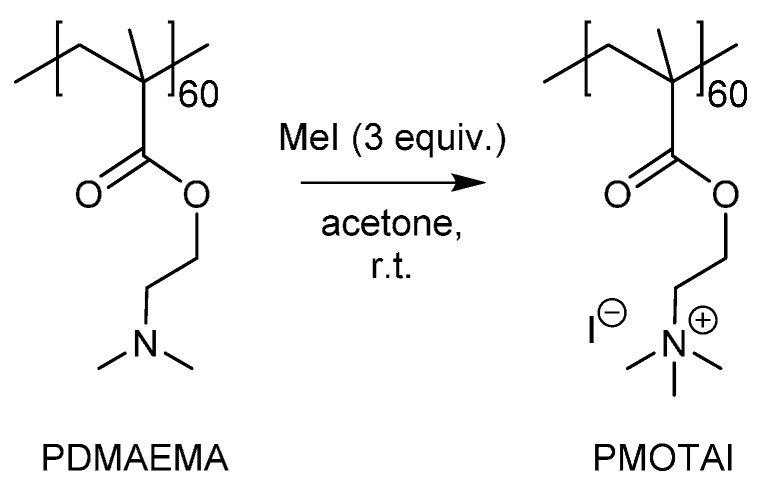
Quaternization of the DMAEMA repeating units of the homopolymer or PDMAEMA_n_-*b*-POEGMA_m_ block copolymers *via* reaction with excess iodomethane in acetone.

PMOTAI and PMOTAI_n_-*b*-POEGMA_m_ were synthesized *via* quaternization of the same batches of polymers bearing DMAEMA repeating units (Figure 1). For example, a flask was charged with the PDMAEMA_60_ homopolymer (2.5 g, 15.9 mmol of repeating units), iodomethane (3 mL, 48.1 mmol, 3 equiv. with respect to DMAEMA repeating units) and acetone (100 mL). Iodomethane was added to the clear pink, stirring polymer solution at room temperature. The flask was sealed and the solution was stirred in the dark (22 h). The product formed as a pale pink precipitate and was separated from the solvent using a centrifuge. After decanting the solvent, the residual acetone was removed by vacuum desiccation with heating (80 °C, overnight). The product was then dissolved in acetonitrile, transferred to aqueous solution and freeze dried. Close to quantitative quaternization of the amine was achieved (97% quaternization by ^1^H NMR, 85.5% yield, *M*_n_ = 17,400 g/mol by ^1^H NMR).

^1^H NMR (500 MHz, D_2_O, protons marked “a”–“e” in SM), chemical shift (δ)/ppm: (–CH_2_–) H^a^ = 1.06; (R–CH_3_) H^b^ = 1.02, 1.14; (–COOCH_2_) H^c^ = 4.51; (–CH_2_–N^+^Me_3_) H^d^ = 3.85; (N^+^(CH_3_)_3_) H^e^ = 3.29.

#### 2.2.2. Preparation of Silica-Polyelectrolyte (S-PE) Precipitate Nanocomposites

Sodium silicate (SS) is used as a precursor for the silica incorporated into S-PE composites. The stock SS solution was prepared from solid sodium silicate pentahydrate (Na_2_SiO_3_·5H_2_O) and deionized water. The true concentration of SS was calculated by potentiometric titration of a (~10 mM) solution with 0.1 M HCl (see SM; Figure S11). Precipitates were prepared by mixing 1:1, 2:1, 2.5:1 and 5:1 molar ratios of SS/polymer. The homopolymer concentration was fixed with respect to the molar concentration of amine repeating units, whether the case be with respect to DMAEMA or MOTAI. When using a block copolymer, the concentration of amine units was fixed using the amine unit mole fraction of the copolymer, as determined by ^1^H NMR. An example procedure is described as follows. Stock solutions of PDMAEMA (50 mM) and aqueous sodium silicate (SS, 100 mM), then deionized water were placed sequentially as listed in a 5 mL plastic Eppendorf tube with a magnetic stirrer bar. The final concentration of amine repeating units was always 10 mM and the concentration of SS was varied as required. The solution was stirred throughout the addition of the three components. The pH of the solution was measured after the addition of water (Initial pH). If no additional water was added, the pH was measured after the addition of SS. Aqueous HCl (0.1 M or 1.0 M) was added with continuous stirring and the pH of the solution was measured a second time. This is the pH at which particle growth is initiated. All compounds were added to the tube using an appropriate mechanical pipette. The final volume of the solution was always 2 mL. The volume of HCl required was estimated in advance of the experiment, by titration of an SS/polymer solution of the appropriate ratio (SM, Figure S13).

The mixture was stirred for 1 h. The solid phase was separated by centrifuge (5 min at 3500 rpm). The supernatant was removed using a syringe fitted with a needle. The remaining solid phase was washed with deionized water (2 mL) and centrifuged a second time (5 min at 3500 rpm). Excess aqueous phase was removed using a syringe and needle. The washed precipitate was then freeze-dried and stored in a refrigerator (3–5 °C). The pH and ratios of the components are summarized in the reported results.

#### 2.2.3. Preparation of Silica-Polyelectrolyte (S-PE) Water-Dispersible Nanocomposites

Mixing and pH measurements were made in the same way as for the preparation of precipitates. Importantly, stable S-PE dispersions were possible only when ≤25 mM initial concentration of sodium silicate was used. Initial Na_2_SiO_3_ concentrations of 10, 20 and 25 mM were used against a 10 mM concentration of amine repeating units. The final volume of the solution was always 2 mL. After the addition of HCl, the pH was measured and the stable dispersion was removed from the tube using a syringe without a needle. The syringe was then equipped with a filter (0.45 μm PVDF membrane). The stable dispersion was filtered from the syringe into a polystyrene cuvette suitable for light-scattering measurements. The cuvette was then sealed with a cap and wrapped with Parafilm. Light scattering size measurements were initiated soon (<10 min) after transfer of the stable dispersion to the cuvette. All DLS data points are presented as an average of at least three measurements. Examples of DLS measurements are presented in the Supplementary Materials (SM, Figures S14–S20). The pH and ratios of the components are summarized in the reported results.

### 2.3. Instrumentation

^1^H NMR spectroscopy was conducted using a Bruker “UltrashieldTM Plus” 500 MHz spectrometer (Bruker, Billerica, MA, USA). Dialysis of synthesized polymers was performed using regenerated cellulose dialysis membranes (CelluSep, Seguin, TX, USA) with appropriate nominal molecular weight limits (3500–4000 kDa and 12,000–14,000 kDa). Dynamic Light Scattering (DLS) and ζ-potential measurements were made using a Zetasizer Nano ZS (Malvern Instruments, Malvern, UK), operating at a wavelength of 633 nm and a back scattering angle of 173° at a temperature of 20 °C. The correlation functions of the scattered light intensity were analyzed using a multi-exponential fit based on the inverse Laplace transform algorithm (Contin). Mean values of unimodal distributions of the hydrodynamic diameter (*d*_h_) were analyzed. Potentiometric measurements of pH were made using a MeterLabTM PHM210 standard pH meter (Radiometer Analytical SAS, Lyon, France) fitted with a pH Electrode (VWR International). Colloidal dispersions and precipitates were prepared in (5.0 mL) Eppendorf Tubes (Eppendorf AG, Hamburg, Germany). Scanning Electron Microscopy (SEM) was conducted using a Hitachi S-4800 SE microscope (Hitachi High-Technologies Europe; Krefeld, Germany). Samples were coated with platinum (4 nm film thickness) under reduced pressure using a Cressington 208 HR Sputter Coater (Cressington Scientific Instruments Ltd., Watford, UK). SEM samples on conductive carbon tape adhered to aluminum sample plates were flushed with argon prior to application of the coating. Size Exclusion Chromatography (SEC) measurements were made using a Waters 2410 refractive index (RI) detector (Waters Corporation, Milford, MA, USA). Waters Styragel columns were connected to a Waters 515 HPCL pump; THF eluent containing 1% TBAB was used at a flow rate of 0.8 mL/min. The system was calibrated using poly(methyl methacrylate) standards: column temp. of 30 °C, detector temp. of 22 °C, and injection volume of 50 μL. Thermogravimetric Anaylsis (TGA) was conducted using a Mettler Toledo TGA 850 (Mettler-Toledo International Inc. Greifensee, Switzerland). A (0.5–1.0 mg) sample was placed in a 70 μL Alumina (Al_2_O_3_) pan and heated from 25 to 800 °C at a rate of 10 °C/min under nitrogen flow of 50 mL/min. Infrared spectra were recorded by means of an Infralum FT-801 spectrophotometer (SIMEX Co. Ltd., Novosibirsk, Russia) in KBr pellets. Ultrafiltration of dispersible S-PDMAEMA particles was performed using a Millipore solvent resistant stirred cell (Merck Millipore, Merck KGaA, Darmstadt, Germany) fitted with an Amicon Ultrafiltration disc membrane (regenerated cellulose, NMWC = 100 kDa) under pressure (400 kPa).

## 3. Results

### 3.1. Water-Stable S-PE Nanocomposites

**Figure 2:**
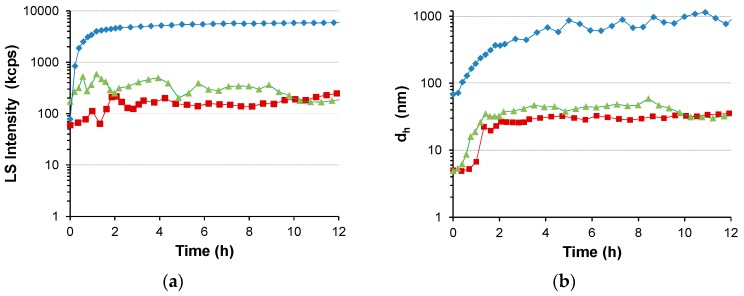
Representative DLS data of S-PDMAEMA_60_ particles prepared at pH 8.0 (blue diamonds ♦), pH 7.4 (green triangles ▲) and pH 6.9 (red squares ■) using a 1:1 feed ratio of SS/PDMAEMA, corresponding to a 10 mM concentration of DMAEMA repeating units, a 1.57 g/L polymer concentration: (**a**) The total intensity of scattered light and (**b**) Mean hydrodynamic diameter of corresponding size distributions (*d*_h_) are shown, as observed over 12 h following acid-triggered initiation of particle growth. Data obtained at a 173° scattering angle using the Zetasizer instrument.

**Figure 3:**
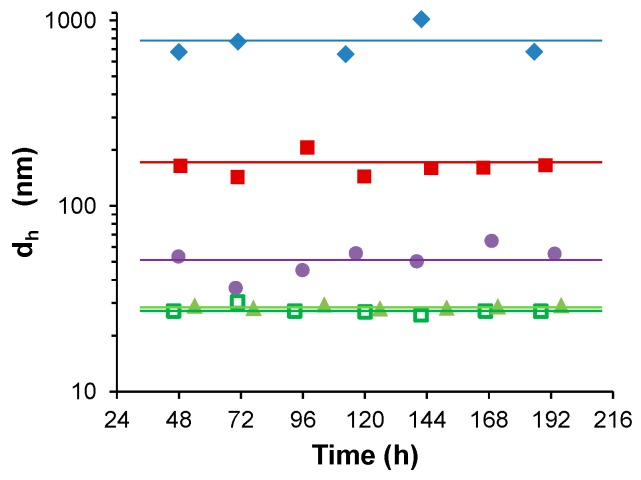
Comparison of stable aqueous colloid dispersions of S-PDMAEMA_60_ particles prepared using 10 mM SS/PDMAEMA (1:1) feed ratio. The initial polymer mass concentration is 1.57 g/L. Particle size shows dependence on the pH at the time of the initiation of particle growth. Particle size by mean hydrodynamic diameter is shown for S-PDMAEMA_60_ particles prepared at pH 5.9 (red squares ■), pH 6.8 (green open squares □), pH 6.9 (green triangles ▲), pH 7.3 (purple circles ●) and pH 8.0 (blue diamonds ♦).

Water-stable S-PDMAEMA_60_ nanoparticles were prepared in the range of pH 5.9–9.6 using a (10 mM) 1:1 feed ratio of SS/PDMAEMA_60_. DLS analysis (Figure 2) of these particles over several days indicated pH dependence of particle size in the cases where no precipitation was observed (pH 6.9, 7.4, 8.0). However, precipitation did occur when particles were prepared outside this pH range (pH <5.9, >9.6). Whereas the particles observed to form at pH 9.6 grew over a long time period and eventually precipitated from solution, those prepared in mildly acidic conditions (pH 5.9) grew rapidly and only partial precipitation from the dispersion occurred (see SM, Figure S14). This was observed as a drop in the total intensity of scattered light around 2 h after initiation of S-PDMAEMA_60_ particle growth. Following the precipitation of the unstable silicate particles, a stable, mono-disperse population of particles was observed to grow over several days. Reaching a mean diameter of 160–170 nm after 7 days, these particles were much larger than those prepared at pH 6.8 and pH 7.3 (*d*_h_ = 30 and 55 nm after 7 days respectively, Figure 3).

The *d*_h_ and zeta (ζ) potential of S-PDMAEMA_60_ prepared at pH 7.5 were measured over a period of 4 days (see SM, Figure S20). The average zeta potential over the entire measurement period was +28 mV. Despite the equimolar ratio of SS and PDMAEMA repeating units, the positive sign of the ζ-potential suggests formation of a polycationic corona on the particle surface that contributes electrostatic stabilization to the S-PE colloid. This ζ is not enough to stabilize the particles completely and it is likely that steric stabilization also has role to play. A good agreement was observed of S-PE *d*_h_ and light scattering intensity of the zeta potential sample, in comparison with a control sample obtained from the same batch of particles. The constant mean and unimodal distribution of *d*_h_, in conjunction with the stable intensity of scattered light throughout the measurement period, demonstrates that S-PDMAEMA_60_ remained stable in the dispersion state under the applied effective voltage of 80 V.

**Figure 4:**
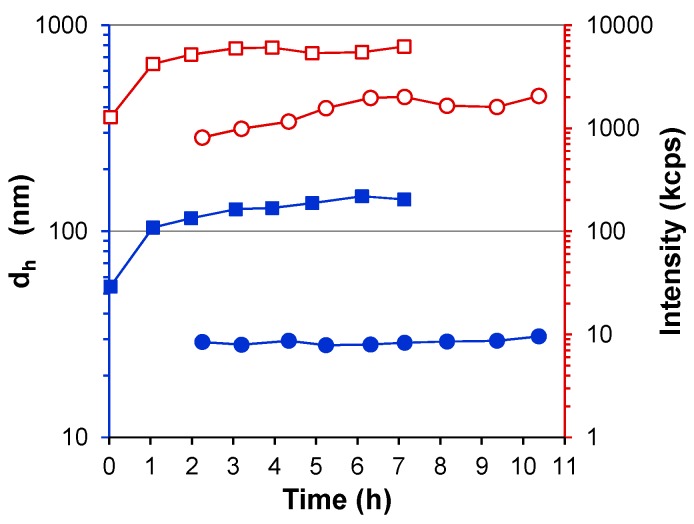
Time-dependent change in the mean hydrodynamic diameter (*d*_h_, nm) of S-PDMAEMA_60_ particles prepared at pH 6.8 using feed ratio of 1:1 SS/PDMAEMA (10 mM SS, filled blue circles ●) and of 2:1 SS/PDMAEMA (20 mM SS, filled blue squares ■) presented as a function of time (h). Here the concentration of DMAEMA repeating units is 10 mM and the polymer concentration is 1.57 g/L. Light scattering intensity (I, kcps) is shown as open red symbols of corresponding shape (○ and □). Data obtained at a 173° scattering angle using the Zetasizer instrument.

Larger S-PDMAEMA_60_ nanocomposites were obtained when using a higher initial concentration of SS at constant pH. The effect of increasing the SS/PDMAEMA ratio was studied at pH 6.8, where particles prepared at ratios of 1:1 and 2:1 were observed over more than 7 days by DLS (Figure 4). S-PDMAEMA_60_ particles of *d*_h_ of 140–150 nm were obtained using a 20 mM concentration (2:1) of SS in the feed, while those prepared from an equimolar (1:1) feed remained stable around a mean *d*_h_ of 30 nm. The same trend was observed for S-PMOTAI_60_ at pH 6.7 (see SM, Figure S16). The upper pH limit of stable colloid preparation falls with greater concentrations of SS in the feed: precipitation of S-PDMAEMA_60_ was observed at pH 9.6 using 10 mM SS, pH 9.3 using 20 mM SS and pH 7.7 using 25 mM SS. Precipitation occurred using 50 mM SS in (5:1) SS/PDMAEMA_60_ mixtures across the entire pH range investigated here (pH 5.5–pH 10.1).

Preparation of 10 mM solutions of amine repeating units from PMOTAI and the block copolymers results in higher polymer mass concentrations (see SM, Table S3). However, these concentrations are sufficiently low to guarantee dilute polymer solutions, wherein no polymer aggregates are observed in the DLS measurements of the polymers in the absence of silicates. The visibility of large polymer aggregates is reduced by decreasing the polymer concentration and eliminated at high scattering angle (θ). An example is provided in the Supplementary Materials (see SM, Figure S9).

**Figure 5:**
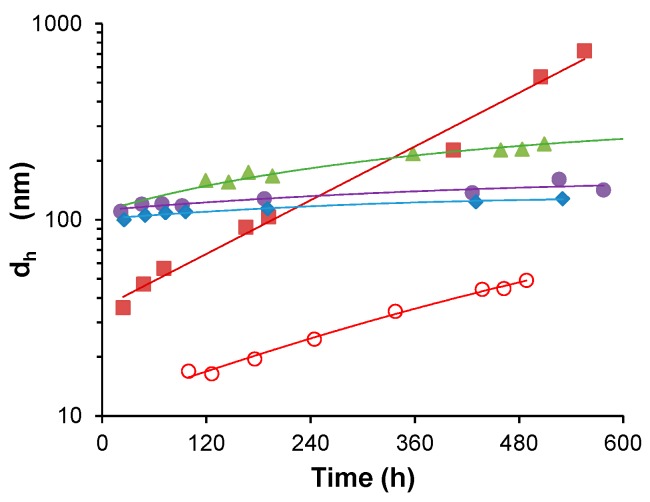
Colloidal dispersions of (1:1) S-PMOTAI particles prepared at pH 6.0 (red circles ○), pH 6.7 (dark red squares ■), pH 7.8 (green triangles ▲), pH 7.9 (purple circles ●) and pH 8.6 (blue diamonds ♦). A constant 10 mM concentration of MOTAI repeating units is used (2.99 g/L). The mean hydrodynamic diameter (*d*_h_, nm) is shown as a function of time (h). Lines are drawn only as a guide for the eye.

Unlike PDMAEMA, the cationic strength of the strong polyelectrolyte PMOTAI is not dependent on pH. The ammonium of the MOTAI repeating unit has a positive charge and an iodide counterion (Figure 1). Increase in particle size was observed as the initiation pH was decreased from pH 8.6 to pH 7.8. S-PMOTAI_60_ particles prepared at pH 8.6, pH 7.9 and pH 7.8 were observed to have *d*_h_ of 120, 160 and 210 nm respectively after 20 days (Figure 5). At pH 6.7, continual growth in the size of particles in dispersion was observed over the same measurement period. S-PMOTAI_60_ prepared at pH 6.0 were unstable: a fraction of the dispersed population of nanocomposites precipitated from solution within 12 h of particle growth initiation. The size of particles shown in Figure 5, for the dispersion of S-PMOTAI_60_ prepared from a pH 6.0 solution, is that of a single population of particles which spontaneously formed after precipitation of the unstable species.

PDMAEMA_60_-*b*-POEGMA_38_ was used to successfully prepare S-copolymer composite dispersions. The contribution of steric stabilization by OEGMA to S-copolymer particles was assessed by DLS in the range pH 6.0–8.1 using a 1:1 SS/PDMAEMA_60_-*b*-POEGMA_38_ feed ratio with respect to 10 mM concentration of DMAEMA repeating units. S-PDMAEMA_60_-*b*-POEGMA_38_ particles prepared at pH 6.0 were *meta*-stable (precipitation occurred after 12 h) but those prepared in the range pH 6.6–8.1 formed stable colloids, as observed by DLS over several days (see SM, Figure S17). These were uniformly smaller than water-stable S-PE nanocomposites prepared in the same range of pH using the PDMAEMA_60_ homopolymer.

**Figure 6:**
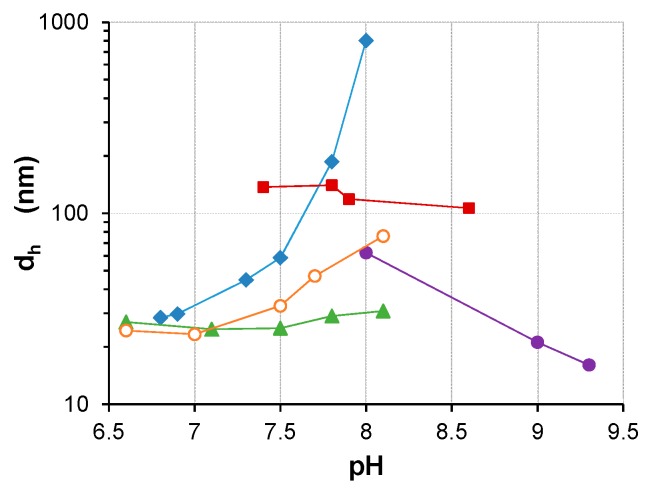
The mean hydrodynamic diameter (*d*_h_, nm) of stable S-PE particles 72 h after particle growth initiation by addition of the appropriate volume of (0.1 M) HCL (see Experimental Section 2.2.3). Each data point is obtained using exponential fit of individual DLS data sets (*i.e.*, *d*_h_ *vs.* time curves) gathered at the corresponding pH: S-PDMAEMA_60_ (blue diamonds ♦), S-PMOTAI (red squares ■), S-PDMAEMA_60_-*b*-POEGMA_38_ (green triangles ▲), S-PMOTAI_60_-*b*-POEGMA_38_ (purple circles ●) and PDMAEMA_300_ (orange circles ○). All S-PE composites were prepared using a 1:1 (10 mM) SS/DMAEMA or SS/MOTAI ratio with respect to the concentration of amine-functional repeating units.

The size (*d*_h_) is presented as a function of the initial solution pH at which S-PE particles were prepared, *via* acid-triggered condensation of SA, as directed by the selected polyelectrolytes PDMAEMA_60_, PDMAEMA_300_, PMOTAI_60_, PDMAEMA_60_-*b*-POEGMA_38_ and PMOTAI_60_-*b*-POEGMA_38_ (Figure 6). Exponential fits were used to estimate the S-PE particle size 72 h after the initiation of particle growth. Analysis of data gathered by DLS revealed that, in the range pH 6.6–8.1 after 24 h, S-PE particle growth in every case had either ceased or slowed to a rate that allows reasonable estimation of particle size. In every case, DLS data points were available close to either side of the time of 72 h, affording good accuracy for the estimation of particle size at the chosen time.

The hydrodynamic diameters of particles plotted on Figure 6 are of unimodal populations of S-PE nanocomposites. Outside the range of pH presented for each dataset, either bimodal distributions or precipitation occurred. In the cases of PDMAEMA_60_ and PDMAEMA_300_, the largest particles were prepared at pH 8.0 and pH 8.1, respectively. However, the higher *M*_w_ PDMAEMA_300_ (at pH 8.1) yielded nanocomposites approximately one order of magnitude smaller than lower *M*_w_ PDMAEMA_60_ (at pH 8.0). The longer dangling chains of the higher molecular weight polymer contribute more effective steric stabilization. Under more basic conditions, weaker electrostatic interactions result in precipitation of S-PDMAEMA, regardless of the homopolymer *M*_w_.

The size of the prepared S-PDMAEMA_60_-*b*-POEGMA_38_ nanocomposites displays little pH dependence. Similarly, the size of S-PMOTAI_60_ nanocomposites varies little in the range of pH 7.4–8.6, although these are all much larger, with *d*_h_ in excess of 100 nm. A single population of S-PMOTAI_60_ particles was successfully prepared at pH 7.4 but under more acidic conditions a bimodal distribution of sizes was observed and, at pH 6.0, some of the S-PMOTAI_60_ particles precipitated from dispersion. S-PMOTAI_60_-*b*-POEGMA_38_ nanocomposites could not be prepared below pH 8.0.

### 3.2. Precipitate S-PE Nanocomposites

Precipitation of S-PDMAEMA_60_ was achieved using 5:1, 2.5:1, 2:1 and 1:1 feed ratios of SS/PDMAEMA. A 10 mM concentration of PDMAEMA_60_ repeating units was used in all cases while the concentration SS was altered from 10 to 50 mM as required. The aqueous stability of silicate under this pH regimen was tested by altering the pH of 50 mM solutions of SS in the range pH 6.0–10.0 by the same method in the absence of polymer, whereupon no precipitation was observed. Therefore, in the SS/Polymer systems, precipitation was induced by action of the polymers. TGA analysis of the precipitate material confirmed the presence of polymer: dynamic analysis from 25 to 800 °C at 10 °C/min revealed the mass percentage of silicate remaining once all the polymer had thermally decomposed (Table 2). Neat sodium silicate (SS) was tested by TGA under identical conditions and showed no change in mass once trapped water had been removed by evaporation during heating from 25 to 150 °C (see SM, Figure S22).

An optimal pH condition is implied by comparison of S-PDMAEMA_60_ precipitate particles prepared using a 5:1 feed ratio (Table 2; 1–6). Of these, the greatest mass percentage was observed for S-PDMAEMA_60_ prepared at pH 7.4 (65.7%). Under more acidic conditions than the optimum pH, association of PDMAEMA with dispersed silicates is limited by the availability of deprotonated Si-OH-groups for electrostatic interactions; under more basic conditions, other interactions are limited by the degree of protonation of the polymer repeating units.

**Figure 7:**
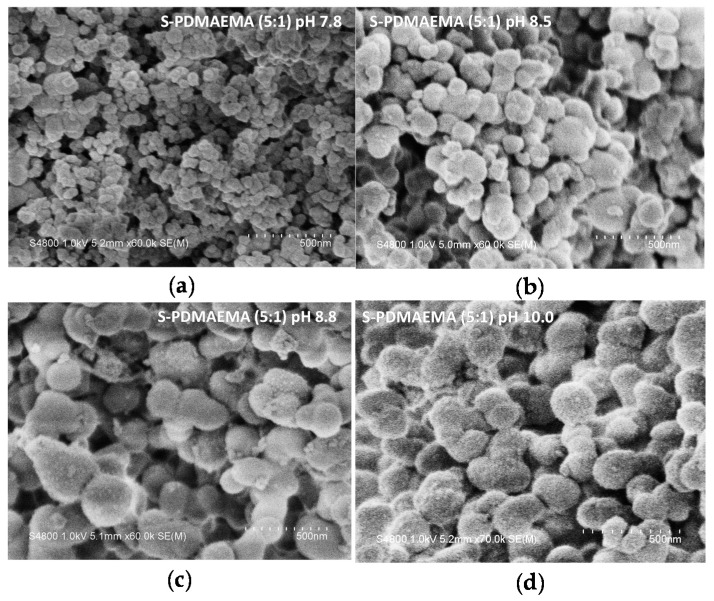
SEM images of S-PDMAEMA_60_ precipitates prepared using a 5:1 feed ratio of SS/PDMAEMA at (**a**) pH 7.8; (**b**) pH 8.5; (**c**) pH 8.8 and (**d**) pH 10.0. Polymer concentration was fixed at 10 mM of repeating units. Frames (**a**)–(**c**) are at 60,000× magnification; frame (**d**) is 70,000× magnification. A 500 nm scale bar is provided within each frame.

**Figure 8:**
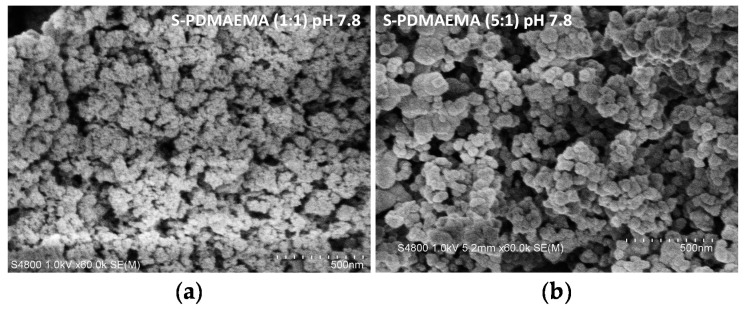
SEM images of water-stable and precipitated composites (60,000× magnification). (**a**) S-PDMAEMA water-stable particles prepared at pH 7.8 using a 1:1 feed ratio of SS and PDMAEMA and extracted by ultrafiltration; (**b**) precipitated S-PDMAEMA particles prepared at the same pH using a 5:1 feed ratio and collected by centrifuge. Both samples were washed with deionized water, isolated by freeze-drying and adhered to electrically conductive carbon tape for SEM imaging.

Scanning Electron Microscopy (SEM) of the S-PDMAEMA_60_ precipitate nanocomposites revealed spherical particles (Figure 7a–d). Samples of S-PDMAEMA which contain greater amounts of silica are those consisting of smaller particles (Table 2; 1–4). The S-PDMAEMA_60_ precipitates prepared at pH 7.8 (61.3% silica) are around 50 nm in diameter and are smaller than those prepared at pH 8.5, pH 8.8 and pH 10, all of which have diameters in the range 150–200 nm. Water dispersible (1:1, pH 7.8) S-PDMAEMA_60_ was also analyzed by SEM (see Figure 8). TGA of these same samples showed (1:1) S-PDMAEMA_60_ contains 65.7% silica by mass and (5:1) S-PDMAEMA_60_ contains 61.3% (Table 2). A fivefold increase in SS concentration does not significantly increase the silica mass fraction in the S-PDMAEMA_60_ composite. Both examples are close to the limit of S-PE silica mass fraction using the specific polymer studied here, regardless of the colloidal stability of the composite.

The water-stable S-PDMAEMA_60_ (1:1, pH 7.8) was observed by DLS to have a mean *d*_h_ of around 190 nm after 72 h (Figure 6). SEM images revealed clusters of S-PDMAEMA_60_ spheres around one quarter of the diameter of those observed by DLS. This disparity in size is attributed to the nature of the sample, its preparation for measurement and the measurement technique. The solid sample observed by SEM is obtained by ultrafiltration of the dispersion, isolation by freeze-drying, then adhesion onto carbon tape. Swelling of the hydrophilic polymer in solution and aggregation of the particles when filtered under pressure can contribute to changes in the apparent size of the composite particles.

## 4. Discussion

Neat SA is monomeric at concentrations of less than 2 mM but forms dimers and polymerizes at higher concentrations into poly(silicic acid) (PSA) colloidal particles. Precipitation of neat silicic acid (SA) does not occur in the range of pH 5.0–pH 10.0 by the method implemented in this work, using a 50 mM concentration of the sodium silicate (SS) precursor. At pH 7.8 in the absence of polymer, SA forms a colloid with *d*_h_ in the range 600–800 nm, which remains stable in dispersion for several days (see SM, Figure S10). This is in good agreement with similar results obtained in the literature [43]. These large particles are difficult to characterize by DLS due to their high polydispersity, and low specific refractive index increment [47].

The specific refractive index increment (dn/dc) of PSA in water is small (dn/dc = 0.06 mL/g) for the scattering of (λ_o_ = 438 nm) visible light [47]. This describes the low intensity of scattered light per unit mass of PSA, as observed by DLS. The value is much larger for the polycation PDMAEMA, however, for which dn/dc = 0.18 mL/g (λ_o_ = 633 nm) in water [45,48]. Physical association of PDMAEMA with PSA results in the formation of particles which scatter more light and are therefore more easily detectable by DLS than neat PSA particles of equivalent mass. This effect is noticeable across the range of pH tested here. At pH 7.8, for example, neat PSA was observed to scatter around 120 kcps while S-PDMAEMA particles prepared at the same pH have a scattering intensity of 7200 kcps, using the same instrumental setup.

The degree of protonation (α) of PDMAEMA and silicates in aqueous solution was determined experimentally by titration of the polymer and the sodium silicate precursor against 0.1 M HCl (see SM, Figure S11). From these titration curves, α was calculated using the Henderson-Hasselbach equation:

pH = pK_a_ + log((1−α)/α) (1)

**Figure 9:**
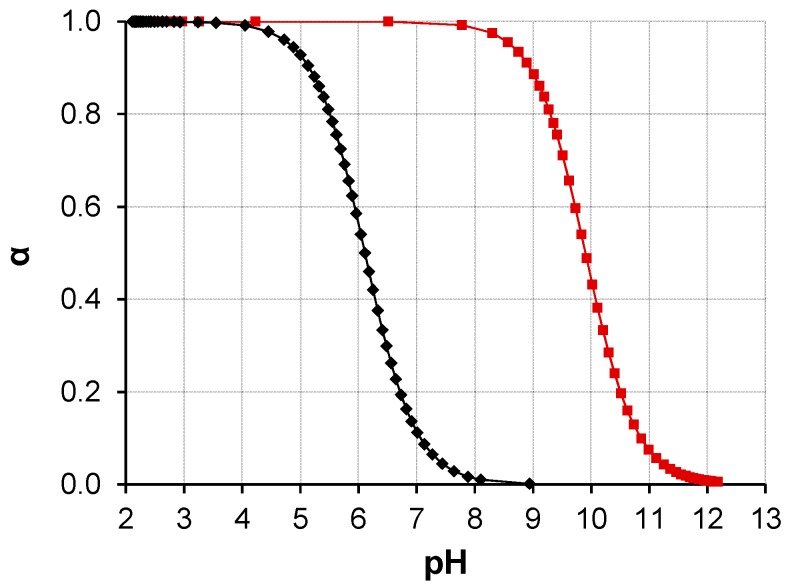
Degree of protonation (α) of PDMAEMA (10 mM of repeating units, 1.57 g/L, black diamonds ♦) and silicate (initially SiO_3_^2−^ from a 10 mM solution of SS, which undergoes hydrolysis to yield SA, red squares ■) solutions as a function of pH, as determined by titration. The pKa (PDMAEMA) = 6.1 and pKa (SA) = 9.9.

Rearrangement for α and plotting against pH illustrates well how PDMAEMA and SS differ in their pH response (Figure 9). When studying PDMAEMA and SS separately, it is clear that the neat silicates are already well protonated before the equivalent (10 mM) concentration of PDMAEMA repeating units has begun to accept protons from the solution. In the region of pH 8–10, very little charging is induced along the PDMAEMA chain. However, in the region pH 6–8, the number of PDMAEMA repeating units which are charged increases rapidly as the pH decreases. When 7 < pH < 8, strong silicate-polymer interaction is expected as both silanol-amine and silanol-ester hydrogen bonding and ~Si–O^−^^+^NHR_2_~ ionic interactions are possible. The hydrogen bonding during particle formation is implied by the IR spectra of the precipitated composites (SM, Figures S23–S25). However, the isoelectric point of silanol groups present on the surface of bulk silica has been reported at pH 2 [26,49], so any conditions at pH > 2 would allow the exchange of protons with the silica surface.

The apparent pKa of PDMAEMA changes in the presence of salts. Titration of PDMAEMA_60_ against HCl was performed in the presence of NaCl and Na_2_SO_4_ (see SM, Figure S12). These are analog for the conversion of SS to SA and the effect this has on ionic strength. Na_2_SO_4_ represents the initial condition of SS in solution (no SA has been formed yet) and NaCl represents the final conditions (all Na_2_SiO_3_ has reacted). The true ionic strength is always between the two extremes. This experiment shows that, although the ionic strength varies during the preparation of the composites, it does not significantly affect the pKa of PDMAEMA. In the presence of 100 mM NaCl, pKa (PDMAEMA) = 7.3 and in the presence of 50 mM Na_2_SO_4_, pKa (PDMAEMA) = 7.6, using a 10 mM concentration of DMAEMA repeating units. The same sample of PDMAEMA_60_ has pKa = 6.1 in the absence of salt (Figure 9). Although the pKa of PDMAEMA is higher in the presence of salts, only a small change is affected by the difference in the −1 and −2 anionic charges. As reported in Table 3, S-PE precipitate composites are obtained upon the addition of acid to a solution containing (10 mM) PDMAEMA_60_ and (50 mM) SS. However, no precipitation of the same homopolymer was observed in the presence of (100 mM) NaCl or (50 mM) Na_2_SO_4_ at room temperature.

Titration of PDMAEMA_60_ against HCl in the presence of SS is a more complex matter. Protonation, hydrolysis and condensation of silicates occur upon addition of strong acid to the solution containing SS. The pH response of the (5:1) SS/PDMAEMA_60_ system, which is initially a solution at pH 12.8, is dominated by the silicates of high dissociation constants (pKa = 11.8 and 9.9)—see SM, Figure S11 and also reference [50]. During titration, all available protons are taken up by silicates until they are fully protonated and equilibrium conditions of silicate condensation have been reached. Only at low pH is the abundance of free protons sufficient to induce charging along the PDMAEMA chain. This is visible in the titration curve of for the (5:1) SS/PDMAEMA_60_ system against HCl, wherein two steps are observed in the plot, for the protonation of the silicates and PDMAEMA, respectively (see SM, Figure S12). The procedure reported in the present work consequently involves the rapid addition of the precise amount of strong acid to achieve the desired pH for the formation of S-PE composites: slow addition of HCl under titration conditions precludes the role of the weak polycation in directing S-PE particle formation at pH > 8.

The association of the polyelectrolytes with aggregate PSA is dependent on the pH of the solution. In S-PE composite formation directed by the weak polycations PDMAEMA_60_ and PDMAEMA_60_-*b*-POEGMA_38_, the pH dependence arises from the degree of charging of the amine groups along the polymer chain and from the pH-dependent aggregation behavior of SA. In the case of PMOTAI, charging of the quaternary ammonium repeating unit is not pH dependent, although the aggregation of SA and the surface charge of PSA particles remain so.

**Figure 10:**
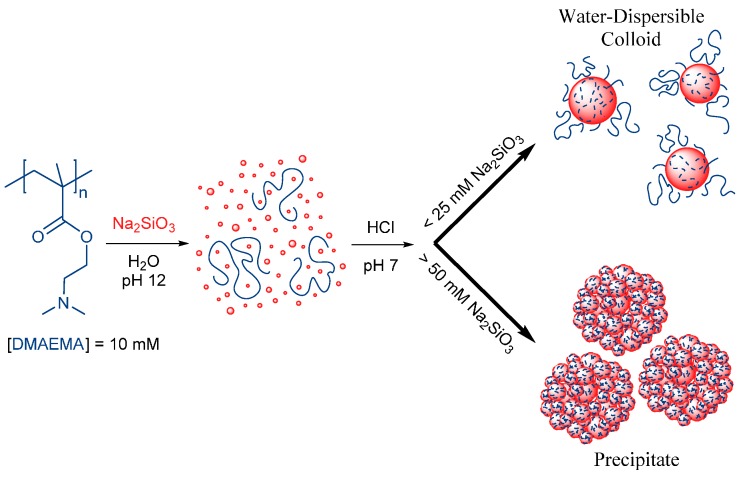
Acid-triggered hydrolysis of sodium silicate (Na_2_SiO_3_) and subsequent polycondensation of silicic acid (Si(OH)_4_) is directed by polycation bearing DMAEMA repeating units. The resultant silica-polyelectrolyte (S-PE) nanocomposites either precipitate or form water-stable (dispersible) particles, depending on the initial concentration of the sodium silicate precursor with respect to the (10 mM) concentration of DMAEMA repeating units.

Using an appropriate molar ratio of SS and amine (DMAEMA) or ammonium (MOTAI) repeating units, either dispersible or precipitate S-PE nanocomposite particles are selectively obtained by the reported method, in the studied pH regime at room temperature (see Figure 10). Water-dispersible colloids of S-PE nanocomposites were obtained using a 10–25 mM concentration of the SS precursor with respect to a 10 mM concentration of cationic DMAEMA or MOTAI polymer repeating units. S-PE precipitate nanocomposites are prepared using 50 mM SS across the studied pH regime or at 10–25 mM SS at higher pH (Table 3).

The ranges of pH presented in Table 3 are those at which particle formation was achieved in this research and are not intended as exhaustive boundary conditions of pH for S-PE particle formation by the reported method. It is demonstrated that in the range pH 6–8 both colloidal and precipitate particles can be obtained with respect to the feed ratio of SS/amine repeating units. S-PDMAEMA_60_ can be prepared when particle growth is initiated in the range pH 6.8–8.0; however, using higher concentrations of the SS precursor lowers the upper limit of pH. When the SS/DMAEMA ratio is 1:1, colloids of S-PDMAEMA_60_-*b*-POEGMA_38_ can be prepared in the same range of pH as for both the PDMAEMA_60_ and PDMAEMA_300_ homopolymers. S-PMOTAI_60_ is prepared in a narrower range, pH 6.0–8.6, but precipitates when using a 2.5:1 SS/MOTAI ratio at the same pH regime.

Even at a 1:1 SS/DMAEMA ratio, using PDMAEMA_60_, precipitation occurs at pH 9.6 (Table 3). Above pH 9, PDMAEMA is practically uncharged and able to interact with silanol groups of PSA via H-bonds. In this case, the number of contacts of a PDMAEMA chain with a single PSA particle is small. One macromolecule can interact with several silica particles and flocculate the dispersion. This effect is already visible as the formation of large yet stable particles at pH 8 (Figure 3).

The PDMAEMA_60_-*b*-POEGMA_38_ block copolymer comprises a bulky POEGMA block to contribute steric stabilization to the S-PE composite in dispersion. However, the POEGMA block is also a physical barrier to the electrostatic charge at the silica-PDMAEMA interface. Moreover, the build-up of osmotic pressure as two S-PDMAEMA_60_-*b*-POEGMA_38_ particles approach each other prevents aggregation of the composites. The growth of S-PDMAEMA_60_-*b*-POEGMA_38_ particles, by condensation of SA onto their charged surface and by their aggregation with PSA and other composites, is thus inhibited by the POEGMA block. This model is supported by the absence of significant pH dependence of the size of S-PDMAEMA_60_-*b*-POEGMA_38_ (Figure 9). The POEGMA block readily acts as a flocculant in the presence of the strong cationic charge of PMOTAI, such that S-PMOTAI_60_-*b*-POEGMA_38_ dispersions could not be prepared at pH < 8.0 by the reported method (Table 3).

## 5. Conclusions

In this work, the acid-triggered condensation of silicic acid from aqueous solution is directed by the weak polycation PDMAEMA, the strong polycation PMOTAI, and their derivative PDMAEMA-*b*-POEGMA and PMOTAI-*b*-POEGMA block copolymers. The resultant silica-polyelectrolyte (S-PE) nanocomposites are prepared under mild conditions of pH and can be selectively obtained as either stable, aqueous dispersions (colloids) or as particles which precipitate from the solution (see Figure 10).

The size of the S-PE colloid particles depends on the mass of the PDMAEMA homopolymer, the strength of the polycation and the presence of the sterically bulky POEGMA block (Figure 6). Dispersions of S-PDMAEMA_60_ are obtained from solutions of the homopolymer and sodium silicate (SS), wherein the molar ratio of SS/PDMAEMA is 1:1–2.5:1 with respect to a 10 mM concentration of DMAEMA repeating units. The size of S-PDMAEMA_60_ increases from *d*_h_ = 30 to 800 nm when the pH at which particle formation is initiated is increased from pH 6.8 to 8.0. The mass percentage of silica in dispersible S-PDMAEMA_60_, prepared at pH 7.8 (*d*_h_ = 200 nm), was 66%. Dispersible S-PMOTAI_60_ nanoparticles do not vary much in size with solution pH (d_h_ = 120–150 nm) and a stable dispersion can only be obtained in a narrower range of pH 7.8–8.6, below which partial precipitation or bimodal size distributions are observed. In the range of pH 6.6–8.1, the higher *M*_w_ PDMAEMA_300_ directs the formation of smaller nanocomposites which vary only slightly in size. The size of S-PDMAEMA-*b*-POEGMA nanoparticles does not vary significantly under the same conditions. Precipitation of S-PE nanocomposites is observed at higher pH than those indicated, or when using greater relative concentrations (25–50 mM) of the SS precursor (see Table 3).

In the range of pH 6–8, the following interactions can take place: (i) electrostatic interactions between the anionic, deprotonated silanol groups of oligomeric silicates and PSA with the protonated (cationic) amine of PDMAEMA and (ii) hydrogen bonding between silanol and the nitrogen lone pair of the amine.

Hydrolysis of sodium silicate provides the *in situ* formation of silicic acid (SA) required for S-PE particle formation. SA is a weak acid (pKa = 9.9) and is well protonated below pH 8 (see Figure 9). However, acid-catalyzed condensation of SA yields oligomers, which have pKa = 6–7 [50]. At close to neutral pH, further condensation of free monosilicic acid proceeds *via* reaction with the ionized silanol groups of the oligomers. Growth of a silica particle proceeds *via* condensation of successive monosilicic acid molecules onto the anionic particle surface.

The amine groups of PDMAEMA carry increasing cationic charge when the solution pH is decreased from pH 8 to pH 6, to a maximum of around 60% (Figure 9). Electrostatic interactions of the weak polycation with the anionic silanol surface of PSA particles results in inter-polyelectrolyte complexation and the release of the low molar mass counterions. Condensation of monosilicic acid onto the composite exterior and aggregation of S-PE particles results in an uneven distribution of the polycation throughout the volume of the S-PE composite. However, the polymer can also cover the particle surface, forming free loops and dangling chains. Concerted steric and electrostatic stabilization of the S-PE particles maintains the observed colloidal state. The dispersible S-PDMAEMA_60_ nanoparticles prepared at pH 7.8 (66% silica by mass) and isolated by ultrafiltration, repeated washing with deionized water and freeze-drying, were revealed by SEM as clusters of spheres.

The stabilizing capacity of PDMAEMA is limited: the polymer begins to act as a flocculant when the relative amount of SA in solution is increased. Small S-PE composites aggregate to form secondary “raspberry-like” particles. The hydrophilic surface-to-mass ratio of these aggregates is insufficient for the stability of the dispersion and results in precipitation of the composite (see Figure 10). SEM of freeze-dried S-PDMAEMA_60_ precipitates revealed polydisperse spherical particles of 50–200 nm in diameter, depending on solution pH at which particle formation was initiated. The mass percentage of bound silica in the precipitated S-PDMAEMA_60_ (45%–65%) and S-PDMAEMA_60_-*b*-POEGMA_38_ (53%–57%) nanoparticles varies in the studied range of solution pH, with a maximum close to neutral conditions (Table 2 and SM Table S4).

The unique contribution of this research is the definition of the conditions of pH under which silica-polyelectrolyte (S-PE) nanocomposites can be prepared. These are selectively obtained as either water-stable dispersions or as precipitates. Furthermore, the size of the colloidal S-PE particles is dependent on the pH at which particle growth was initiated. The S-PE composites are obtained as spherical nanostructures, harvested from aqueous solutions of the selected precursors under mild conditions.

## Figures and Tables

**Table 1:** Amounts of DMAEMA, PDMAEMA, OEGMA, AIBN initiator and CPA chain transfer agent used in the synthesis of PDMAEMA and PDMAEMA_n_-*b*-POEGMA_m_.

Polymers	Ratio (M:I:CTA)	DMAEMA (mmol)	DMAEMA (g)	OEGMA (mmol)	OEGMA (g)	AIBN (mmol)	AIBN (mg)	CPA (mmol)	CPA (mg)	PDMAEMA (mmol)	PDMAEMA (mg)
**PDMAEMA_60_**	**50:0.1:1.0**	63.61	10.00	-	-	0.13	21.7	1.28	358.0	-	-
**PDMAEMA_60_-*b*-POEGMA_38_**	**60:0.1:1.0**	-	-	4.73	2.00	0.01	1.5	-	-	0.08	745.0
**PDMAEMA_60_-*b*-POEGMA_100_**	**120:0.1:1.0**	-	-	9.46	4.00	0.01	1.5	-	-	0.08	742.0

**Table 2:** Thermogravimetric Analysis (TGA) of S-PDMAEMA_60_ precipitates except 9 *, which is water-dispersible S-PDMAEMA_60_ isolated by ultrafiltration. The pH at which S-PDMAEMA_60_ formation was initiated is shown, as well as the feed ratio of SS/PDMAEMA. (For examples of the TGA plots, see SM Figure S21).

	Ratio SS:PDMAEMA	pH	Silica Mass Residue (%)
1	5:1	10.0	49.5
2	5:1	8.8	46.4
3	5:1	8.5	55.5
4	5:1	7.8	61.3
5	5:1	7.4	65.7
6	5:1	6.8	62.3
7	2.5:1	7.7	51.1
8	2:1	9.3	46.0
9 *	1:1	7.8	65.7
10	1:1	9.6	57.1

**Table 3:** Colloid (water-dispersible) and precipitate S-PE composites, as prepared in this work under different conditions of pH, with respect to the initial ratio of SS/amine repeating units. The concentration of amine repeating units in all cases is 10 mM. Four components were used in the preparation of all S-PE composites: sodium silicate, polymer, hydrochloric acid and deionized water. Colloids are defined as those S-PE composites which remained stable in aqueous dispersion for at least 7 days. The total initial volume of each solution was 2 mL. The pH of the solution was adjusted by addition of the appropriate amount of (0.1 or 1.0 M) HCl. A range of pH values are shown to illustrate the maximum and minimum values of pH in a series.

Polymer	Polymer Conc. (g/L)	SS Conc. (mM)	Ratio (SS:amine units)	pH	Result
**PDMAEMA_60_**	1.57	10	1:1	6.8–8.0	colloid
				9.6	precipitate
		20	2:1	6.8–7.8	colloid
				9.3	precipitate
		25	2.5:1	6.1–7.5	colloid
				7.7	precipitate
		50	5:1	6.1–10.0	precipitate
**PDMAEMA_300_**	1.57	10	1:1	6.6–8.1	colloid
**PDMAEMA_60_-*b*-POEGMA_38_**	2.15	10	1:1	6.6–8.1	colloid
		50	5:1	6.6–8.5	precipitate
**PMOTAI_60_**	2.99	10	1:1	6.0–8.6	colloid
		20	2:1	6.7	colloid
		25	2.5:1	6.5–6.9	precipitate
**PMOTAI_60_-*b*-POEGMA_38_**	4.79	10	1:1	7.5	precipitate
				8.0–9.3	colloid

## Supplementary Materials

The supplementary materials can be found at www.mdpi.com/2073-4360/8/3/96/s1.

## Abbreviations

The following abbreviations are used in this manuscript:

AIBN	2,2′-azobisisobutylnitrile (or 2,2′-azobis(2-methylpropionitrile))
ATRP	atom-transfer radical-polymerization
CPA	4-cyanopentanoic acid dithiobenzoate
CTA	chain transfer agent
DLS	dynamic light scattering
EBiB	ethyl α-bromoisobutyrate
IR	infrared spectroscopy
LCPAs	long-chain polyamines
NMR	nuclear magnetic resonance
OEGMA	oligo(ethylene glycol) methyl ether methacrylate
PDMAEMA	poly(2-(dimethylamino)ethyl methacrylate)
PE	polyelectrolyte
PMOTAI	poly(methacryloxyethyl trimethylammonium iodide)
PSA	poly(silicic acid)
RAFT	reversible addition fragmentation chain-transfer polymerization
SA	silicic acid (≡orthosilicic acid, monosilicic acid)
SEC	size exclusion chromatography
SEM	scanning electron microscopy
SM	supplementary materials
S-PE	silica-polyelectrolyte (nanocomposite)
SS	sodium silicate (sodium water glass)
TBAB	tetra-*n*-butylammonium bromide
TEOS	tetraethyl orthosilicate
TGA	thermogravimetric analysis
THF	tetrahydrofuran
TMOS	tetramethyl orthosilicate
