# Supplementary material for: Water-Dispersible Silica-Polyelectrolyte Nanocomposites Prepared via Acid-Triggered Polycondensation of Silicic Acid and Directed by Polycations
Source: Polymers (Basel). 2016 Mar 22;8(3):96. doi: 10.3390/polym8030096 (PMC6432522; doi:10.3390/polym8030096)
Supplement: Supplementary file 1 [file polymers-08-00096-s001.pdf]

Supplementary Materials: Water-Dispersible Silica-Polyelectrolyte Nanocomposites Prepared via Acid-Triggered Polycondensation of Silicic Acid and Directed by Polycations

Philip Overton, Elena Danilovtseva, Erno Karjalainen, Mikko Karesoja, Vadim Annenkov, Heikki Tenhu and Vladimir Aseyev

1. Polymer Synthesis

Table S1. Monomer conversion and reaction yield (by mass).

	Conversion ^{1}H NMR (%)	Yield (%)
**PDMAEMA$_{60}$**	69.7	76.9
**PMOTAI$_{60}$**	-	85.5
**PDMAEMA$_{60}$-*b*-POEGMA$_{38}$**	62.3	66.1
**PMOTAI$_{60}$-*b*-POEGMA$_{38}$**	-	98.6
**PDMAEMA$_{60}$-*b*-POEGMA$_{100}$**	83.2	88.7
**PMOTAI$_{60}$-*b*-POEGMA$_{100}$**	-	96.2

Table S2. Summary of synthesized polymers; conversions, yields, polydispersity (PDI), M_n and M_w are shown. Results obtained by Nuclear Magnetic Resonance (NMR) spectroscopy and Size Exclusion Chromatography (SEC) are quoted as such. PDI is given as calculated from the SEC molecular mass distribution. The degree of polymerization (DP) is shown as theoretical ($DP_{Theor.}$) and observed DP (M_n/m) values. $DP_{Theor.}$ = ([monomer]/[CTA]) × conversion. $DP_{Theor.}$ for chain extension of PDMAEMA with OEGMA was calculated using DP (M_n/monomer molecular mass) of $PDMAEMA_{60}$. M_n by ^{1}H NMR was calculated by end group analysis; values marked ([a]) are calculated using the M_n (^{1}H NMR) of the $PDMAEMA_{60}$ macro-CTA, which underwent chain extension with OEGMA. M_n calculated by ^{1}H NMR was used in preference over M_n by SEC where possible. PMOTAI-*b*-POEGMA block copolymers were synthesized via quaternization of the corresponding PDMAEMA-*b*-POEGMA block copolymer. Included is the OEGMA macro-monomer used in the synthesis of the block copolymers.

	**Monomer molecular mass (g/mol)**	**M_w SEC (g/mol)**	**M_n SEC (g/mol)**	**M_n ^{1}H NMR (g/mol)**	**PDI SEC (M_w/M_n)**	**$DP_{Theor.}$**	**DP (M_n/m)**	**$DP_{Theor.}$/DP**
**OEGMA**	-	-	-	423	1.32	-	-	
**$POEGMA_{30}$**	-	26,600	24,100	12,800	1.11	-	30	
**$PDMAEMA_{60}$**	157.21	9,200	8,200	9,400	1.12	34.9	60	0.58
**$PMOTAI_{60}$**	299.12	-	-	17,400	-	-	-	
**$PDMAEMA_{60}$-*b*-$POEGMA_{38}$**	-	25,300	21,600	-	1.17	97.2	104	0.94
**$PMOTAI_{60}$-*b*-$POEGMA_{38}$**	-	-	-	34,500 [a]	-	-	-	
**$PDMAEMA_{60}$-*b*-$POEGMA_{100}$**	-	51,950	44,050	-	1.18	141.2	159	0.89
**$PMOTAI_{60}$-*b*-$POEGMA_{100}$**	-	-	-	63,200 [a]	-	-	-	

1.1. Reversible Addition-Fragmentation Chain Transfer (RAFT) and Atom Transfer Radical Polymerisation (ATRP) Synthesis of Poly(2-(dimethylamino)ethyl methacrylate) (PDMAEMA)

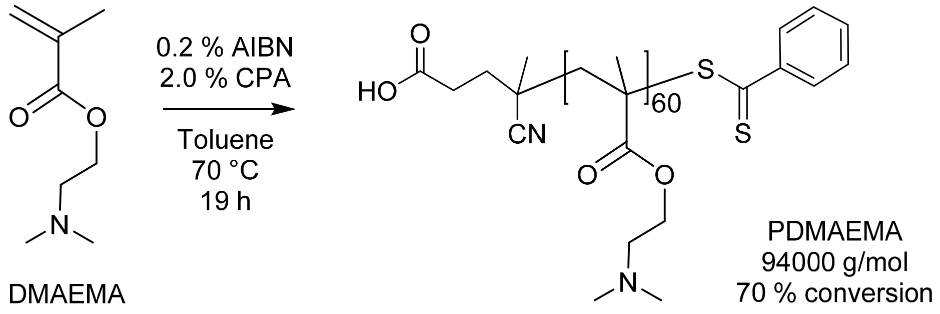

Figure S1. RAFT synthesis of $PDMAEMA_{60}$. Mole percentages of AIBN initiator and CPA chain transfer agent are shown with respect to DMAEMA.

2,2′-Azobis(2-methylpropionitrile) (AIBN) (0.021 g, 0.128 mmol), 4-cyanopentanoic acid dithiobenzoate (CPA) (0.358 g, 1.281 mmol) and DMAEMA (10.011 g, 63.679 mmol) were dissolved in distilled toluene (3 mL) and placed in a 25 mL round bottom flask with a magnetic stirrer bar. The flask was fitted with a stopcock adapter then sealed with grease and Parafilm. The regents formed a clear red solution and were degassed under vacuum by freeze-thawing with liquid nitrogen. The flask was re-pressurized with nitrogen gas and placed in a 70 °C oil bath to initiate the reaction. The flask was heated at 70 °C with stirring (19 h) and the reaction was finally quenched by exposing the reaction mixture to air and submerging the flask in liquid nitrogen. At this point, a conversion sample for NMR analysis was taken.

The product was precipitated from the reaction mixture into cold hexane (500 mL total) and a second time from acetone into cold hexane. The second precipitate was dissolved in the minimum amount of acetonitrile (20 mL) and concentrated by rotary evaporation. The resulting solution was diluted with water (40 mL) and dialyzed against water (4 L, 3 days, water changed 4 times). The coral-coloured product was isolated by freeze-drying (69.7% conversion by ^1H NMR, 76.9% yield by mass, M_n = 9400 g/mol by ^1H NMR end group analysis, M_w = 9200 g/mol by SEC, M_w/M_n = 1.12). Number of repeating units (^1H NMR) = 60.

^1H NMR (500 MHz, D_2O) protons marked "a"–"e" (supporting information), chemical shift (δ)/ppm: (–CH_2–) H^a = 1.854; (R–CH_3) H^b = 0.857, 1.060; (–$COOCH_2$) H^c = 4.101; (–CH_2–NMe_2) H^d = 2.689; ($RN(CH_3)_2$) H^e = 2.274.

Also used in this investigation was $PDMAEMA_{300}$, synthesized using ATRP. The reaction flask was charged with 6.5780 g (41.8 mmol) of DMAEMA, 0.0166 g (0.168 mmol) of CuCl, 0.0056 g (0.0417 mmol) of $CuCl_2$ and 2 mL of acetone. The flask was bubbled with nitrogen for 50 min. A separate solution of 0.0408 g (0.209 mmol) of EBiB and 0.0377 g (0.210 mmol) of PMDETA in 2 mL of acetone were bubbled with nitrogen for 25 min. The reaction flask was moved to an oil bath at 50 °C and the solution containing EBiB and PMDETA was transferred to the reaction flask via a nitrogen-flushed syringe. The reaction was allowed to proceed at 50 °C for five hours, after which the reaction was stopped by sudden cooling with liquid nitrogen and exposure to the atmosphere. At this point, a sample for determining the conversion of the reaction was taken. The reaction mixture was diluted with THF and eluted through a column of Al_2O_3 in top of SiO_2 with THF. The solution was concentrated by evaporation and filtered through 1 μm glass fibre filter. The polymer was purified with two precipitations from acetone to hexane and further purified by dialysis against water. The product was isolated by freeze-drying (86.6% conversion, M_n = 47,700 g/mol and M_w = 61,000 g/mol by SEC, M_w/M_n = 1.28).

1.2. RAFT Synthesis of PDMAEMA$_n$-b-POEGMA$_m$ Copolymers by Chain Extension of PDMAEMA

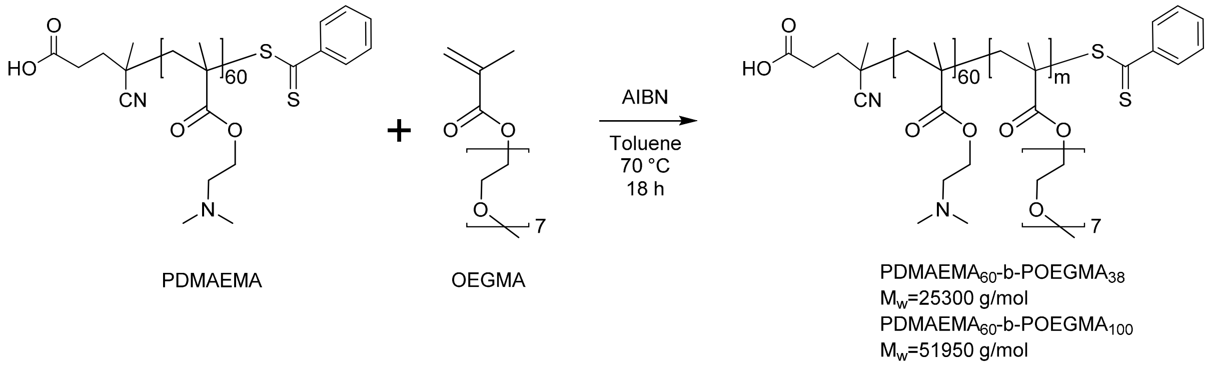

Figure S2. RAFT chain extension of PDMAEMA$_{60}$ with the OEGMA macro-monomer. Two PDMAEMA$_n$-*b*-POEGMA$_m$ copolymers (n = 60 and m = 38 or 100) were prepared. RAFT Z-terminal PDMAEMA was employed as a CTA.

AIBN (1.5 mg, 9.13 × 10^{-6} mol), PDMAEMA$_{60}$ (745 mg, 7.91 × 10^{-5} mol) and OEGMA (2.00 g, 4.73 × 10^{-3} mol) were dissolved in toluene (10 mL) and placed in a 50 mL round bottom flask. Synthesis was carried out as with PDMAEMA. The reaction time was 18 h. The block copolymer was precipitated twice from hexane, dialyzed against water (4 L, 3 days, water changed 4 times) and then freeze dried to obtain the purified PDMAEMA$_{60}$-*b*-POEGMA$_{38}$ product (62.3% conversion by ^{1}H NMR, 66.1% yield by mass, M_w = 25,300 g/mol by SEC, 57.5% DMAEMA by Mass, 67% DMAEMA by ^{1}H NMR).

PDMAEMA$_{60}$-*b*-POEGMA$_{38}$ ^{1}H NMR (500 MHz, D_2O) protons marked "a"-"e" (supporting information), chemical shift (δ)/ppm: (–CH_2–) D^a, O^a = 1.914; (R–CH_3) D^b, O^b = 0.908, 1.074; (–$COOCH_2$) D^c, 1st O^c = 4.155; (–CH_2–NMe_2) D^d = 2.739; $O^{c,d}$ = 3.687; (RN(CH_3)$_2$) D^e = 2.329; (RO–CH_3) O^e = 3.369.

A second PDMAEMA$_{60}$-*b*-POEGMA$_{100}$ block copolymer was prepared in the same way using twice the amount of OEGMA (4.00 g, 9.46 mmol). The product was obtained at 83.2% conversion by ^{1}H NMR, 88.7% yield by mass, M_w = 51,950 g/mol by SEC, 21.4% DMAEMA by Mass, 39% DMAEMA by ^{1}H NMR.

1.3. Quaternization of PDMAEMA: Synthesis of poly(methacryloxyethyl trimethylammonium iodide) (PMOTAI)

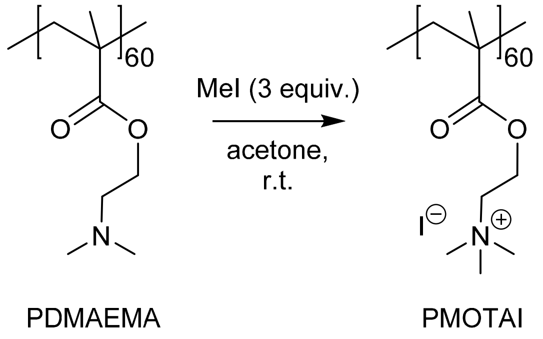

Figure S3. Quaternization of DMAEMA repeating units by reaction with excess iodomethane.

The PDMAEMA$_{60}$ homopolymer (2.5 g, 15.9 mmol of repeating units), iodomethane (3 mL, 48.1 mmol, 3 equiv.) and acetone (100 mL) were placed in a flask. The PDMAEMA was dissolved separately in 20 mL of the total volume of acetone and transferred to the round bottom flask using a syringe. The iodomethane was added to the clear pink, stirring PDMAEMA solution at room temperature. The flask was sealed and the solution was stirred in the dark (22 h). The product formed as a pale pink precipitate and was separated from the solvent using a centrifuge. Additional acetone

(2 × 25 mL) was used to rinse the flask. After decanting the solvent, the residual acetone was removed by vacuum desiccation with heating (80 °C, overnight). The product was then dissolved in acetonitrile, transferred to aqueous solution and freeze dried. Near-quantitative quaternization of the amine was achieved (97% quaternization by ^{1}H NMR, 85.5% yield, M_n = 17,400 g/mol by ^{1}H NMR).

^{1}H NMR (500 MHz, D_2O) protons marked "a"–"e" (supporting information), chemical shift (δ)/ppm: (–CH_2–) H^a = 1.063; (R–CH_3) H^b = 1.023, 1.135; (–$COOCH_2$) H^c = 4.506; (–CH_2–N^+Me_3) H^d = 3.852; ($N^+(CH_3)_3$) H^e = 3.285.

1.4. Quaternization of PDMAEMA$_n$-b-POEGMA$_m$: Synthesis of PMOTAI$_n$-b-POEGMA$_m$ and block copolymers

The same method was used as for $PDMAEMA_{60}$, however quaternization did not induce precipitation of the product polymers. At the end of the reaction time, unreacted iodomethane and the acetone solvent were removed by rotary evaporation. The product polymer was then dissolved in the minimum amount of acetonitrile and concentrated by rotary evaporation. Water (20 mL) was then added directly to the flask and the residual acetonitrile was removed by further rotary evaporation. The aqueous product was transferred to a 40 mL sample vial and the flask was washed with additional water (2 × 5 mL). The washings were added to the sample vial. The product was isolated by freeze-drying. $PMOTAI_{60}$-*b*-$POEGMA_{38}$: quantitative quaternization, 98.6% yield, M_n = 34,500 g/mol. $PMOTAI_{60}$-*b*-$POEGMA_{100}$: quantitative quaternization, 96.2% yield, M_n = 63,200 g/mol.

2. ^{1}H NMR Spectra

2.1. PDMAEMA

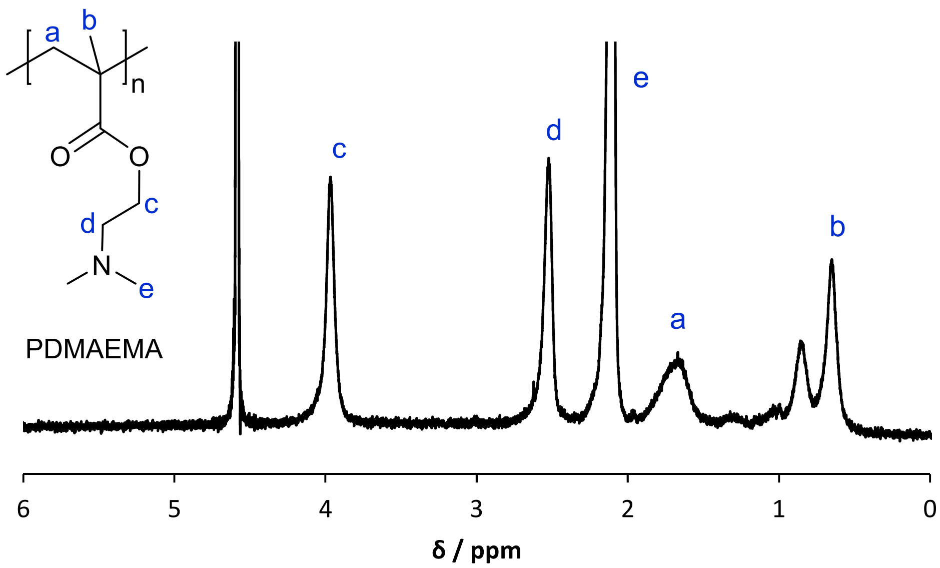

Figure S4. 1H NMR spectrum of PDMAEMA.

^{1}H NMR (500 MHz, D_2O) protons (H) marked "a"–"e", chemical shift (δ)/ppm: H^a = 1.854; H^b = 0.857, 1.060; H^c = 4.101; H^d = 2.689; H^e = 2.274

69.7% conversion by ^{1}H NMR, 76.9% yield by mass, M_n = 9400 g/mol by ^{1}H NMR, M_w = 9200 g/mol by SEC.

2.2. PDMAEMA$_n$-b-POEGMA$_m$ Copolymers

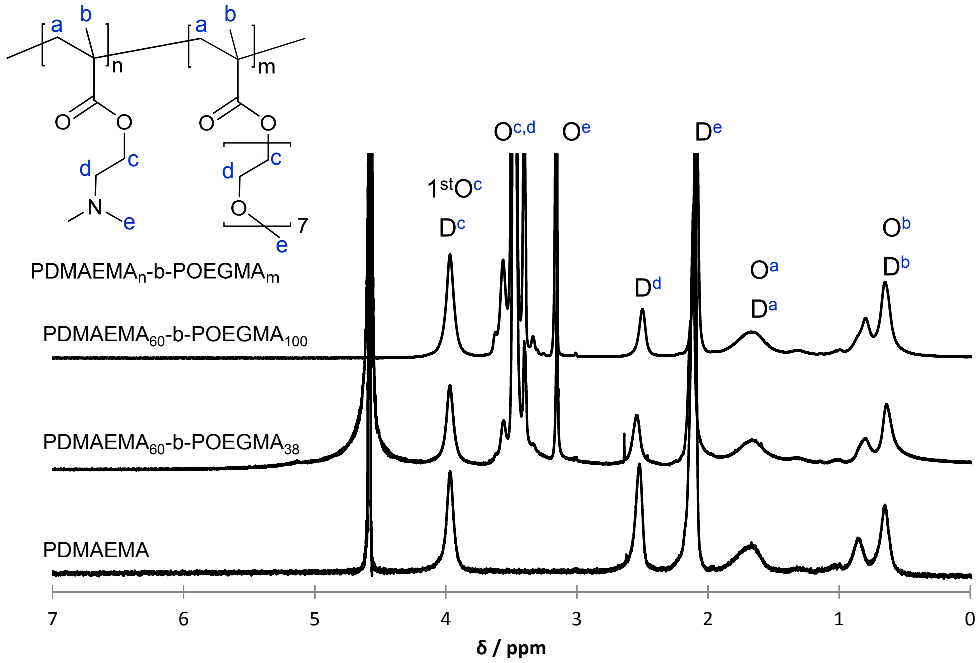

Figure S5. 1H NMR spectra of PDMAEMA and PDMAEMA$_n$-*b*-POEGMA$_m$.

^{1}H NMR (500 MHz, D_2O) protons marked "a"-"e" for DMAEMA (D) and OEGMA (O) repeating units, chemical shift (δ)/ppm: D^a, O^a = 1.914; D^b, O^b = 0.908, 1.074; D^c, 1st O^c = 4.155; D^d = 2.739; O^{c,d} = 3.687; D^e = 2.329; O^e = 3.369.

Block Copolymer 1: PDMAEMA$_{60}$-*b*-POEGMA$_{38}$ 62.3% conversion by ^{1}H NMR, 66.1% yield by mass; SEC: M_w = 25,300 g/mol, 57.5% DMAEMA by mass.

Block Copolymer 2: PDMAEMA$_{60}$-*b*-POEGMA$_{100}$ 83.2% conversion by ^{1}H NMR, 88.7% yield by mass; SEC: M_w = 51,950 g/mol, 21.4% DMAEMA by mass.

2.3. PMOTAI

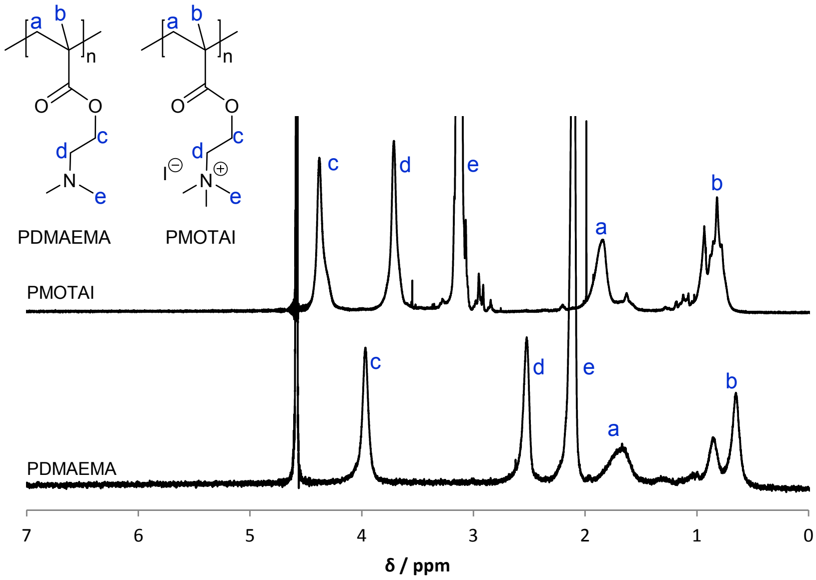

Figure S6. 1H NMR spectra of PDMAEMA and PMOTAI.

PMOTAI: ^{1}H NMR (500 MHz, D_2O) protons (H) marked "a"-"e", chemical shift (δ)/ppm: H^a = 1.063; H^b = 1.023, 1.135; H^c = 4.506; H^d = 3.852; H^e = 3.285.

A total of 100% conversion, 96.9% yield, M_n = 17,900 g/mol by H^1 NMR.

2.4. PMOTAI$_n$-b-POEGMA$_m$ Copolymers

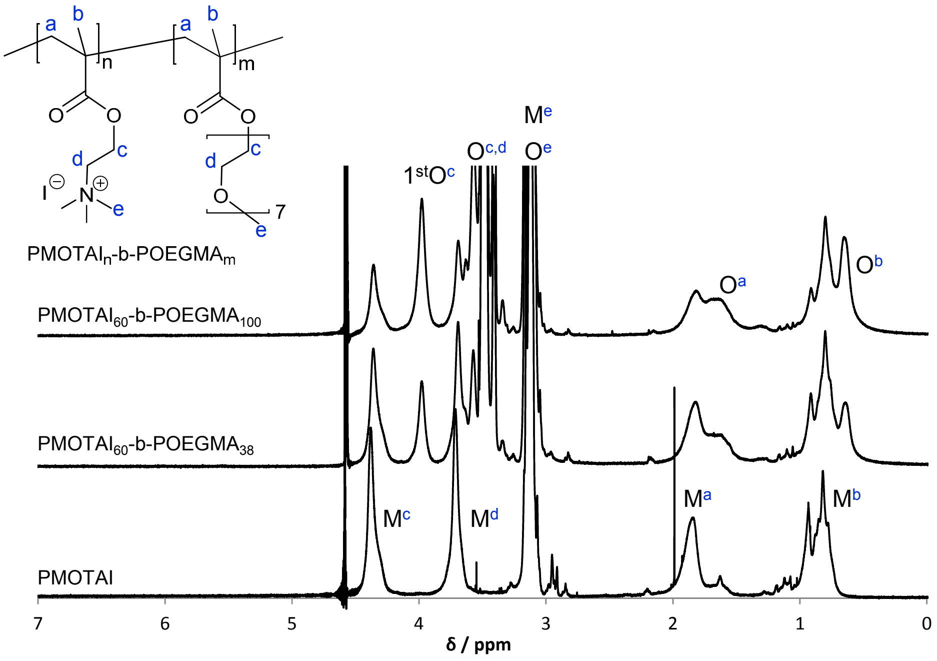

Figure S7. 1H NMR spectra of PDMAEMA and PMOTAI$_n$-*b*-POEGMA$_m$.

^{1}H NMR (500 MHz, D_2O) protons marked "a"-"e" for MOTAI (M) and OEGMA (O) repeating units, chemical shift (δ)/ppm: M^a = 2.02; O^a = 1.87; M^b= 1.02, 1.31; O^b = 0.88; M^c = 4.50; 1st O^c = 4.13; M^d = 3.37; $O^{c,d}$ = 3.65; D^e, O^e = 3.28.

PMOTAI$_{60}$-*b*-POEGMA$_{38}$: 100% conversion, 98.6% yield, M_n = 33,800 g/mol by H^1 NMR.

PMOTAI$_{60}$-*b*-POEGMA$_{100}$: 100% conversion, 96.2% yield, M_n = 60,450 g/mol by H^1 NMR.

3. Size Exclusion Chromatograms of Polymers

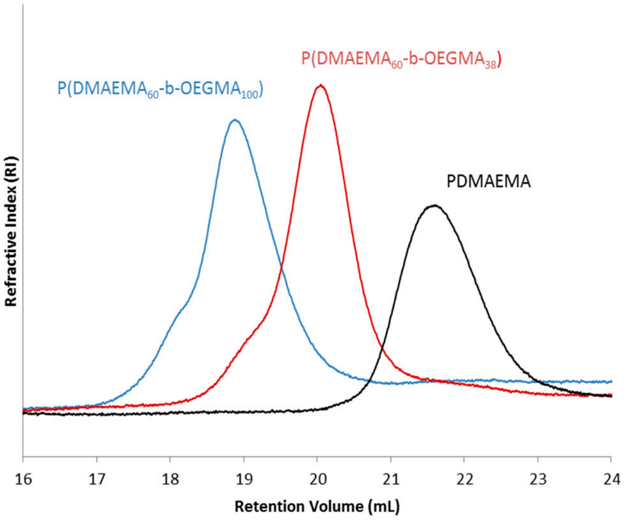

Figure S8. SEC chromatograms of PDMAEMA (black -), PDMAEMA$_{60}$-*b*-POEGMA$_{38}$ (red -) and PDMAEMA$_{60}$-*b*-POEGMA$_{100}$ (blue -). The eluent was THF (containing 1% *v*/*v* TBAB) with 0.8 mL/min flow rate.

4. Preparation of S–PE Composites

4.1. Titration of PDMAEMA against HCl in the Presence of Salt

Stock solutions of PDMAEMA (50 mM), NaCl (200 mM), Na_2SO_4 (100 mM) and Na_2SiO_3 (100 mM) were prepared using deionized water and left stirring overnight before use. Each of these solutions was titrated against 0.1 M HCl with stirring. Potentiometric measurements of pH were made upon stabilization of the pH probe following each addition of acid in ~ 60 s intervals.

4.2. Precipitates

Precipitates were prepared by mixing 1:1, 2:1, 2.5:1 and 5:1 ratios of SS:polymer. The polymer concentration was fixed with respect to the concentration of amine repeating units, whether the case be with respect to DMAEMA or MOTAI. When using a block copolymer, the concentration of amine units was fixed using the amine unit mole fraction of the copolymer as determined by ^{1}H NMR. An example procedure is described as follows. Stock solutions of PDMAEMA (50 mM of repeating units) and aqueous sodium silicate (SS, 100 mM), followed by deionized water were placed sequentially as listed in a 5 mL Eppendorf® tube with a magnetic stirrer bar. The concentration of amine repeating units was always 10 mM and the concentration of SS was varied as required. The solution was stirred throughout the addition of the three components. The pH of the solution was measured after the addition of water (Initial pH). If no additional water was added, the pH was measured after the addition of SS. Aqueous HCl (0.1 M or 1.0 M) was added with continuous stirring and the pH of the solution was measured for a second time (Test pH[a]). All compounds were added to the tube using an appropriate mechanical pipette. The final volume of the solution was always 2 mL.

The mixture was stirred for 1 hour, after which the pH was measured for a third time (Test pH[b]). The solid phase was separated by centrifuge (5 min at 3500 rpm). The supernatant was removed using a syringe fitted with a needle. The remaining solid phase was washed with deionised water (2 mL) and centrifuged a second time (5 min at 3500 rpm). Excess aqueous phase was removed using a syringe and needle. The washed precipitate was then freeze-dried and stored in a refrigerator (3–5 °C). The pH and ratios of the components are summarized in the reported results.

4.3. Water-stable Dispersions

Mixing and pH measurements were made in the same way as for the preparation of precipitates. Importantly, stable S-PE dispersions were possible only when ≤25 mM initial concentration of sodium silicate was used. Initial Na_2SiO_3 concentrations of 10, 20 and 25 mM were used against a 10 mM concentration of amine repeating units. After the addition of HCl, the pH was measured and the stable dispersion was removed from the tube using a 2 mL syringe without a needle. The syringe was then equipped with a filter (0.45 μm PVDF membrane). The stable dispersion was filtered from the syringe into a poly(styrene) cuvette suitable for light-scattering measurements. The cuvette was then sealed with a cap and wrapped with Parafilm. Light scattering size measurements were initiated soon (<10 min) after transfer of the stable dispersion to the cuvette. All DLS data points are presented as an average of at least three measurements. The pH and ratios of the components are summarized in the reported results (see the main article, Table 3).

5. Dispersions

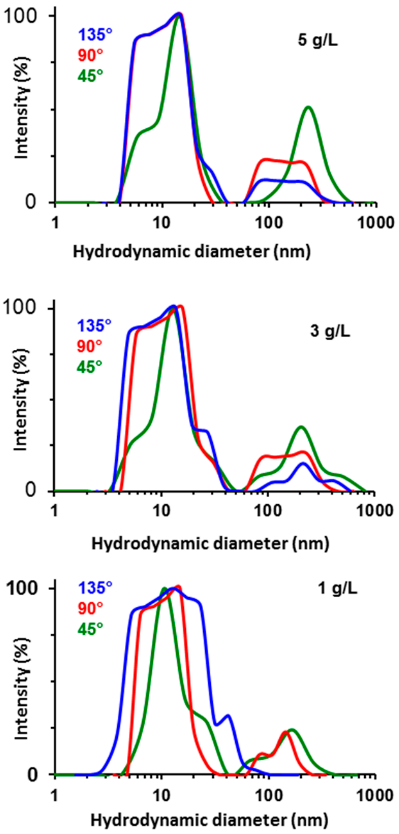

Figure S9. Dynamic Light Scatting (DLS) analysis of $PMOTAI_{60}$-*b*-$POEGMA_{100}$. Correlation functions were fit for 5 g/L (**top**), 3 g/L (**middle**) and 1 g/L (**bottom**) solutions of the block copolymer in 0.5 M NaCl. DLS measurements were carried out at 45° (green lines), 90° (red lines) and 135° (blue lines) scattering angles. Correlation functions fit to the obtained data confirmed the presence of aggregate polymer species. The visibility of larger aggregates is reduced with increasing scattering angle and eliminated by low polymer concentration. DLS measurements of polymer were made using a Brookhaven Instruments BI-200SM goniometer, BI-9000AT digital correlator and a coherent Sapphire 488-100 CDRH laser operating at a wavelength of 488 nm. The temperature was maintained at 20 °C throughout all of the DLS measurements using a Lauda RC 6 CP thermostatic bath. Scattering angles of 45°, 90° and 135° were used. The correlation functions of the scattered light intensity were analysed using a multi-exponential fit based on the inverse Laplace transform algorithm (Contin).

Table S3. Overlap concentration (c *) of selected polymers calculated using the mean R_h determined by DLS CONTIN analysis (θ = 90°) of the 5 g/L polymer in 0.5 M NaCl solution (see Figure S9 for details), except * PMOTAI: DLS (θ = 173°) of 2.99 g/L PMOTAI in the Malvern Zetasizer backscattering instrument. All estimates are based on homopolymers under ideal conditions. For comparison, also shown is the mass concentration of the polymers when a 10 mM solution of amine (DMAEMA or MOTAI) repeating units is prepared. Polymer M_n is quoted as determined by ^{1}H NMR, SEC, or as calculated using the corresponding unquaternized polymer as a reference. Full molecular mass data are given in the Supplementary Materials Table S2.

	M_n (g/mol)	R_h (nm)	C * (g/L)	Mass Conc. (g/L) of 10 mM amine repeating units
**$PDMAEMA_{60}$**	9400 by NMR	2.81	168.3	1.57
**$PDMAEMA_{60}$-*b*-$POEGMA_{38}$**	21,600 by SEC	3.29	298.0	2.15
*** $PMOTAI_{60}$**	17,400 calc.	3.45	168.0	2.99
**$PMOTAI_{60}$-*b*-$POEGMA_{100}$**	63,200 calc.	7.58	57.51	7.92

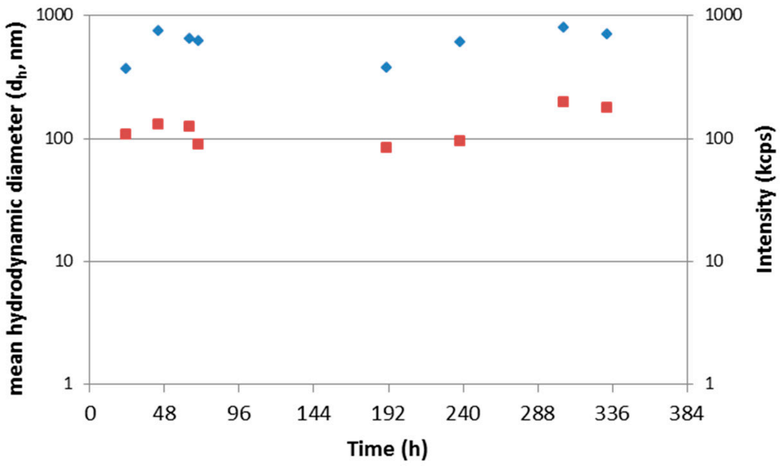

Figure S10. Mean size of SA aggregates prepared from (10 mM) SS *in situ*. A polydisperse population was prepared in deionised water acidified to pH 7.8 by (0.1 M) HCl. The mean size by particle hydrodynamic diameter (nm, blue diamonds ♦) and derived count rate (kcps, red squares ■) are shown.

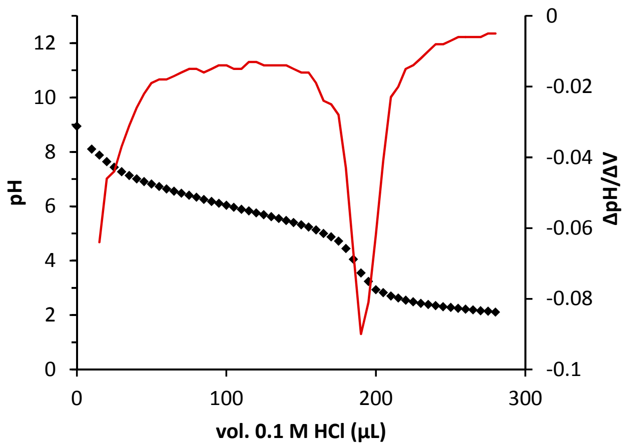

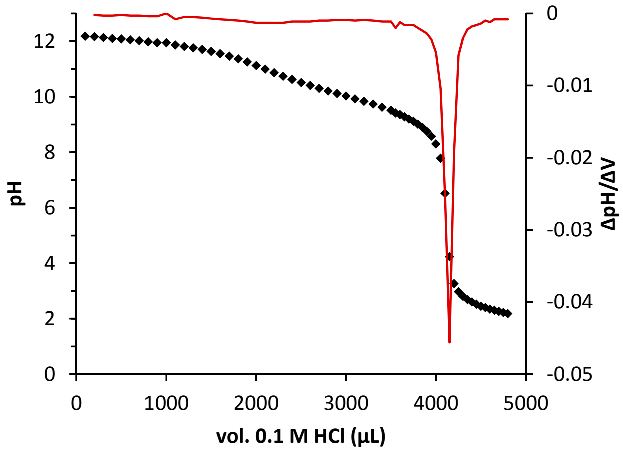

Figure S11. Titration curves of PDMAEMA (10 mM, 1.57 g/L, 2 mL, **top**) and sodium silicate (10 mM, 20 mL, **bottom**) against HCl (0.1 M). The pH (black diamonds ♦) and ΔpH/ΔV (red lines —) of the solutions are shown. The calculated pKa $_{\text{PDMAEMA}}$ = 6.1 and pKa $_{\text{SS}}$ = 9.9.

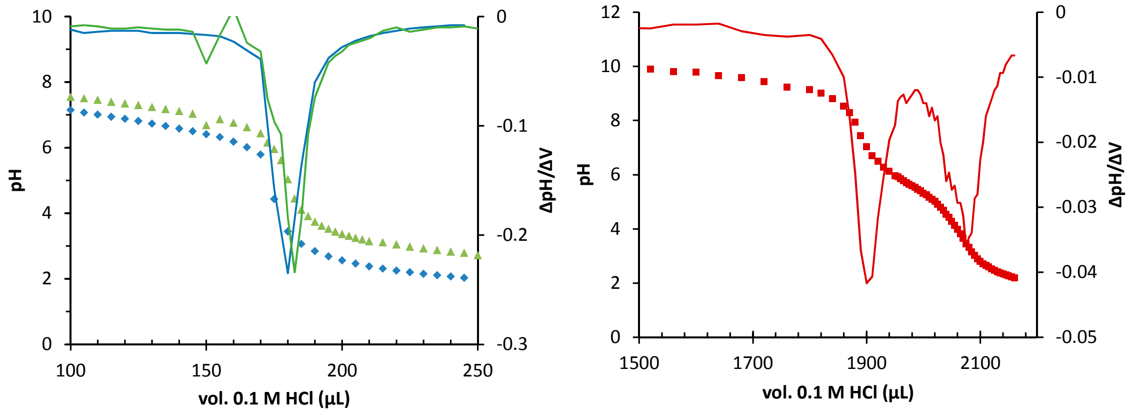

Figure S12. Titration of PDMAEMA (10 mM of repeating units, 1.57 g/L) in the presence of (100 mM) NaCl (**left**), (50 mM) Na_2SO_4 (left) and (50 mM) Na_2SiO_3 (**right**) against 0.1 M HCl. The initial volume of each solution was 2 mL. The ionic strength of the solutions was fixed using different ratios of salt: (10:1) NaCl/PDMAEMA (blue diamonds ♦, pKa = 7.3), (5:1) Na_2SO_4/PDMAEMA (green triangles ▲, pKa = 7.6) and (5:1) Na_2SiO_3/PDMAEMA (red squares ■). ΔpH/ΔV of each data set is shown by straight lines of the corresponding colour.

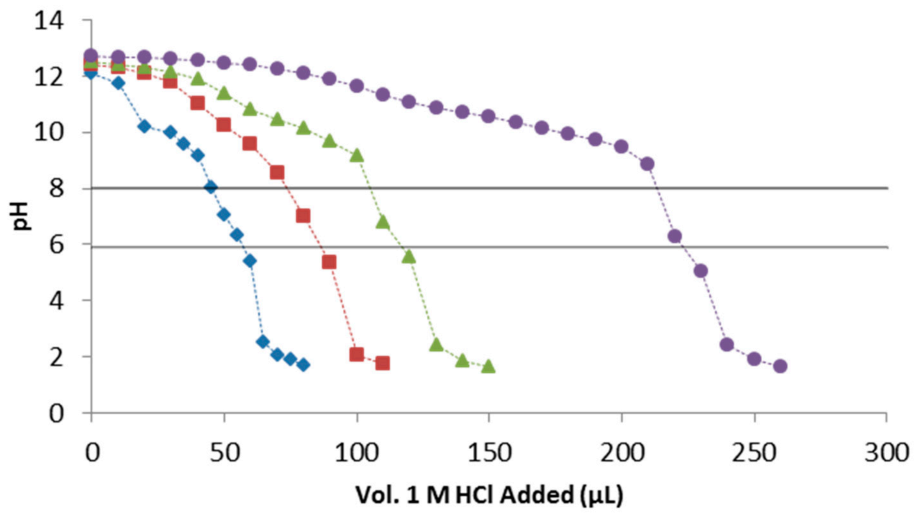

Figure S13. Titration of 2 mL solutions of 1:1 (blue diamonds, ♦), 2:1 (red squares, ■), 2.5:1 (green triangles, ▲) and 5:1 (purple circles, ●) SS/PDMAEMA against 1 M HCl. A 10 mM concentration of DMAEMA repeating units was used (1.57 g/L). The range of stable S-PDMAEMA dispersions prepared from 1:1 SS/PDMAEMA (pH 5.9–pH 8.0; solid lines) is shown.

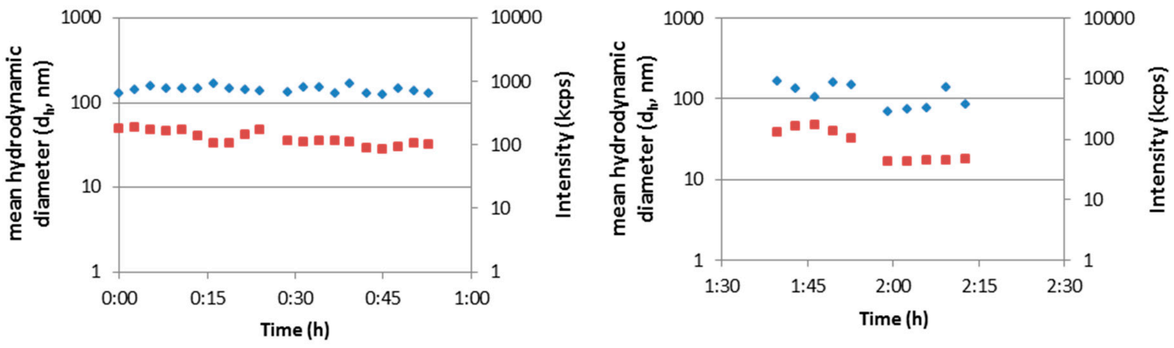

Figure S14. SS/PDMAEMA (1:1, 10 mM) feed ratio S-PDMAEMA particles prepared at pH 5.9 observed over 1h after particle growth initiation (**left**) and during precipitation of unstable population (**right**). The initial polymer mass concentration was 1.57 g/L. The mean hydrodynamic diameter (nm, blue diamonds ♦) and the total intensity of scattered light (kcps, red squares ■) are shown. Decrease in intensity after 1:45 h corresponds to precipitation of particles and a decrease in the number of particles in aqueous dispersion.

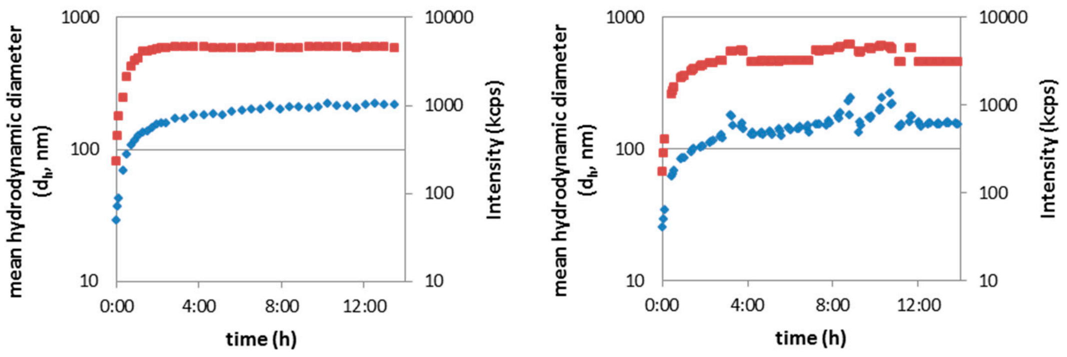

Figure S15. S-PDMAEMA particles prepared at SS/PDMAEMA 2:1 (**left**, [SS] = 20 mM, pH 7.8) and 2.5:1 (**right**, [SS] = 25 mM pH 7.5) ratios corresponding to 10 mM concentration of DMAEMA repeating units, a 1.57 g/L polymer concentration. Plots show a stable (left) and a meta-stable (right) dispersion observed over 12 hours. The mean size by particle diameter (blue diamonds ♦) and the DCR (red squares ■) are shown.

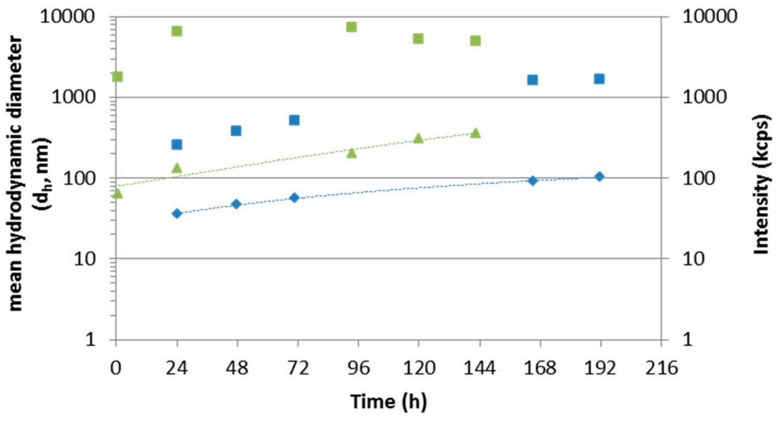

Figure S16. Mean size by hydrodynamic diameter (d_h, nm) of particles in colloidal dispersions of S-PMOTAI prepared at pH 6.7 using 2:1 (green triangles, ▲) and 1:1 (blue diamonds, ♦) feed ratios of SS/PMOTAI are shown as a function of time (h). The derived count rate (kcps) of each sample is plotted (corresponding green and blue squares; ■, ■). Lines are drawn as a guide for the eye.

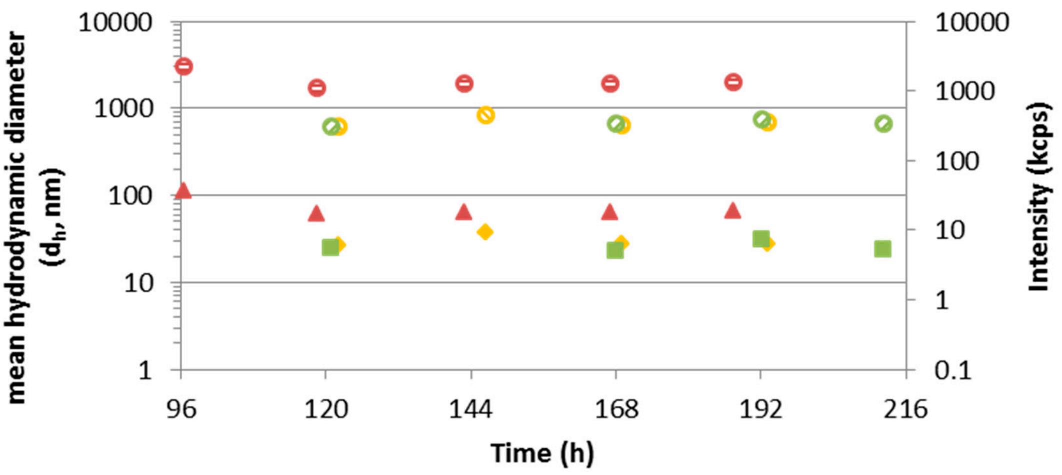

Figure S17. The size by mean hydrodynamic diameter, d_h (nm), of S-PDMAEMA$_{60}$-*b*-POEGMA$_{38}$ particles prepared at pH 6.2 (red triangles, ▲), pH 6.6 (yellow diamonds ♦) and pH 7.1 (green squares, ■) as a function of time (h). The derived count rate (kcps) of each sample is plotted (pH 6.2, ⊖; pH 6.6, ⊘; pH 7.1, ⊘).

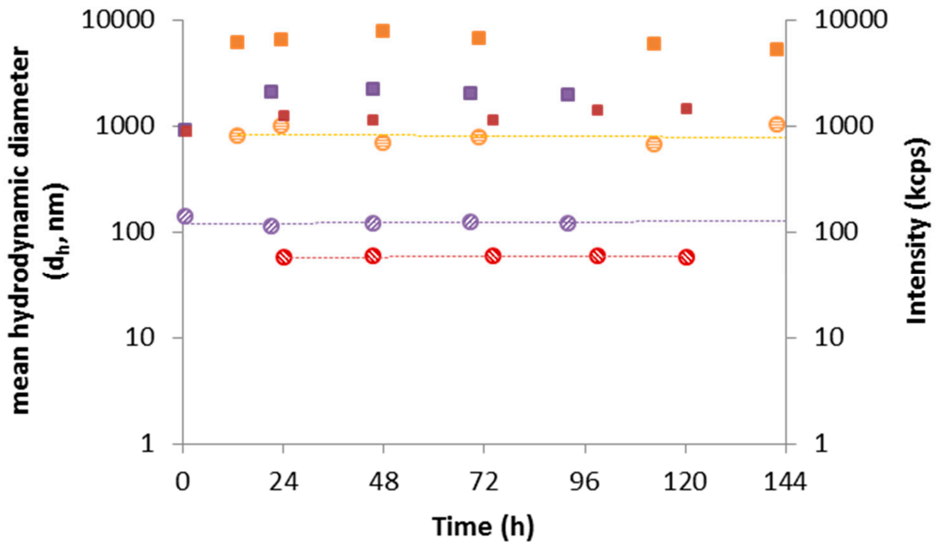

Figure S18. The mean size by hydrodynamic diameter (d_h, nm) of S–PE particles prepared using PDMAEMA (orange, ⊖), PMOTAI (purple, ⊘) and PMOTAI$_{60}$-*b*-POEGMA$_{38}$ (red, ⊘) at pH 7.9–8.0 as a function of time (h). The derived count rate (kcps) of each sample is shown by squares of the corresponding colour (■, ■, ■). Lines are drawn as a guide for the eye.

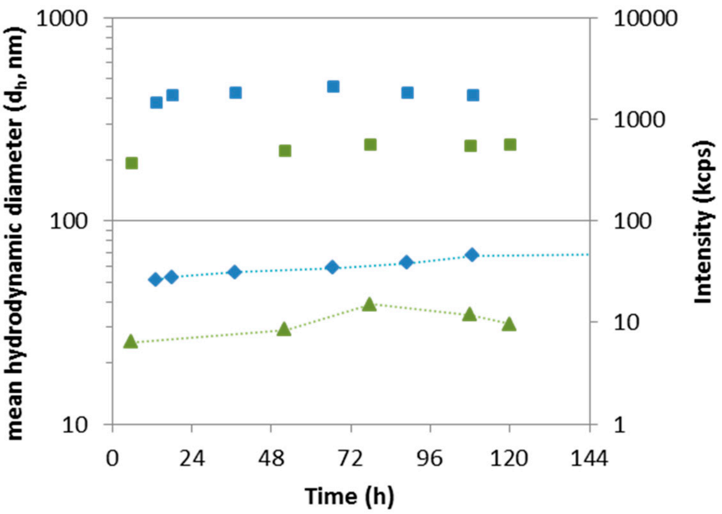

Figure S19. Mean hydrodynamic diameter (d_h, nm) as a function of time (h) for S-PDMAEMA$_{60}$ (blue diamonds ♦) and S-PDMAEMA$_{300}$ (green triangles ▲) particles prepared at pH 7.5. The total intensity of scattered light (kcps) of each sample is shown (squares of the corresponding colour; ■, ■).

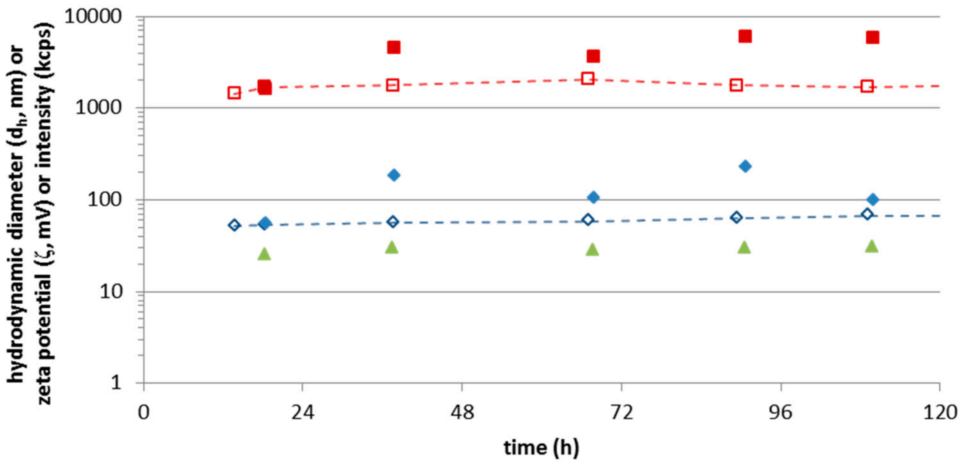

Figure S20. The hydrodynamic diameter (d_h, nm, ♦, ◊), zeta potential (ζ, mV, ▲) and total intensity of scattered light (kcps, ■, □) of S-PDMAEMA particles prepared at pH 7.5 from a 1:1 ratio of SS/PDMAEMA. The initial concentration of the SS precursor was 10 mM; the concentration of DMAEMA repeating units was 10 mM, corresponding to a 1.57 g/L polymer solution. The zeta potential, light scattering intensity and particle hydrodynamic diameter (▲, ♦ and ■ respectively) of the sample in a zeta cell are compared with a control cuvette containing a sample originating from the same S-PDMAEMA dispersion batch (◊, □). Effective voltage = 80.0 V (± 0.3 V), mean zeta potential (ζ, ▲) over the entire measurement period = 28 mV.

6. Thermogravimetric Analysis of Precipitates

Table S4. Thermogravimetric Analysis (TGA) of S-PDMAEMA$_{60}$-*b*-POEGMA$_{38}$. The pH at which S-PE formation was initiated is shown. The 5:1 feed ratio of SS/DMAEMA repeating units was used.

	pH	Silica mass residue (%)
1	6.6	53.5
2	8.5	54.0
3	7.2	56.4
4	8.0	55.6

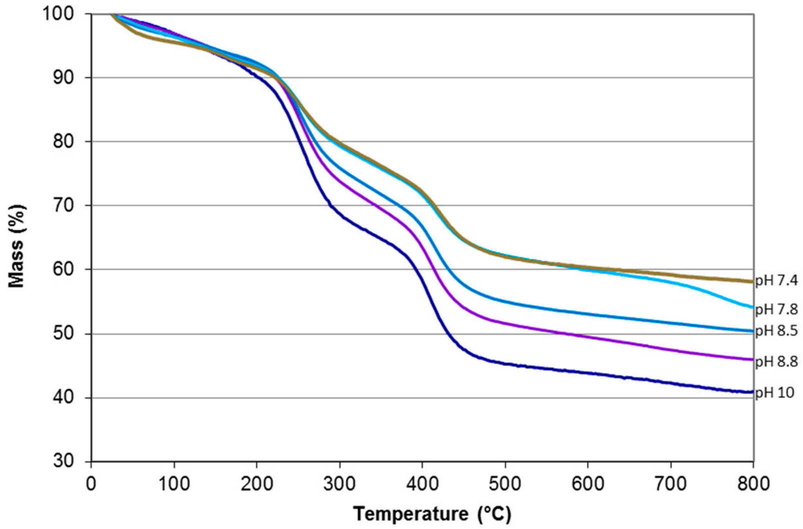

Figure S21. Thermogravimetric Analysis (TGA) of precipitates obtained of S-PDMAEMA$_{60}$ composite particles. Precipitates were prepared using a 5:1 SS/PDMAEMA feed ratio at pH 10 (dark blue line), pH 8.8 (purple line), pH 8.5 (blue line), pH 7.8 (light blue line) and pH 7.4 (green line). A constant heating rate was applied (10 °C/min) from 25–800 °C to samples of 0.8–1.0 mg mass.

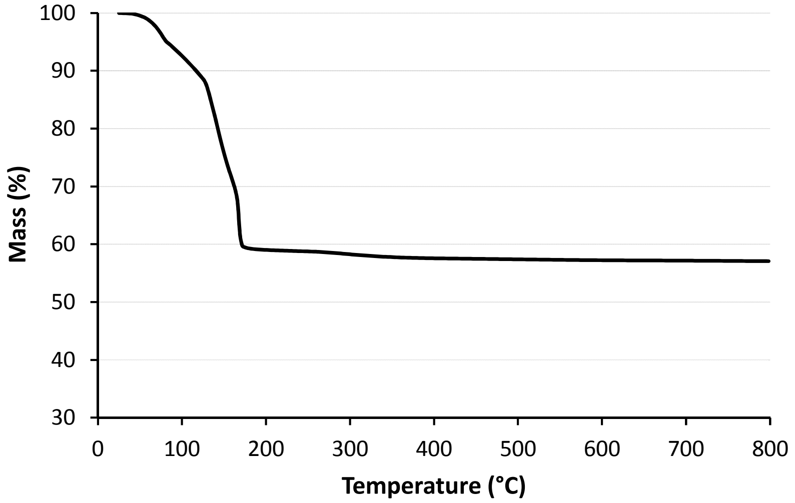

Figure S22. Thermogravimetric Analysis (TGA) of neat sodium silicate pentahydrate ($Na_2SiO_3{\cdot}5H_2O$) from 25 to 800 °C at a rate of 10 °C/min under N_2 gas flow of 50 mL/min. Little change in the mass is observed after the water contained in the sample has evaporated in the region 80–150 °C. No change in sample mass is observed after 400 °C.

7. Analysis of Precipitates by Infrared Spectroscopy

Nature of the S-PDMAEMA interaction was tested by means of infrared spectroscopy (IR), (SM, Figures S23–S25). IR analysis of PDMAEMA and S-PDMAEMA (obtained using experimental conditions described below each figure) reveal vibration bands of the carbonyl group (1730 and 1155 cm^{-1}) and methylene and methyl groups (1470 cm^{-1}) in their spectra. Precipitates show an absorbance at 1080 cm^{-1} typical for Si–O–Si fragments and a vibration band at 990 cm^{-1} typical for ~Si–OH [51]. The PDMAEMA spectrum (black line) was subtracted from the spectrum of S-PDMAEMA (green line). These two spectra were normalized to the absorbance maximum at 1730 cm^{-1}. The result of the subtraction (blue line) is compared with the spectrum of pure silica precipitated from aqueous solution (red line). An increase in relative intensity of the ~Si–OH band in the composite and its shift from 965 (pure silica) to 990 cm^{-1} suggests a higher number of the silanol groups in the S-PDMAEMA composite. This may be a result of hydrogen bonding interactions between the silanol functions of PSA and the amine function of PDMAEMA during particle formation. Similar interactions between cationic macromolecules and silica have been reported [42,43].

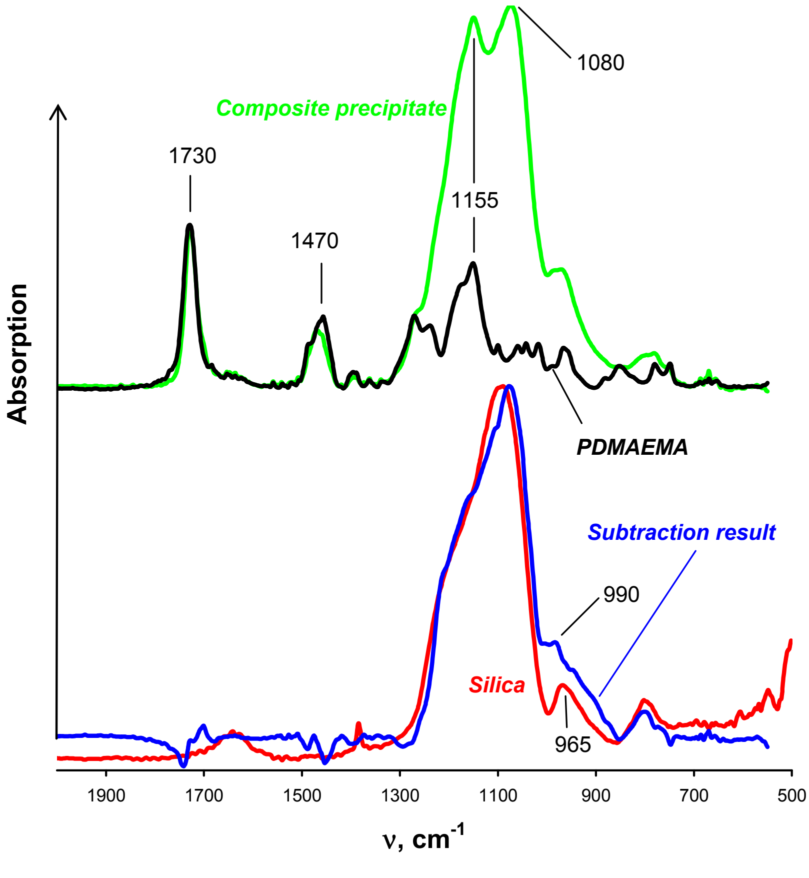

Figure S23. FTIR spectra of PDMAEMA (black line -), pure silica (red line -), S-PDMAEMA composite (green line -) and the result of subtraction of the PDMAEMA spectrum from the composite spectrum subtraction (blue line -): initial SS:DMAEMA = 3:1 (30 mM SS:10 mM DMAEMA) and pH 9.0. Pure silica was obtained by precipitation from 50 mM sodium silicate with 1.0 M HCl at pH 7.0.

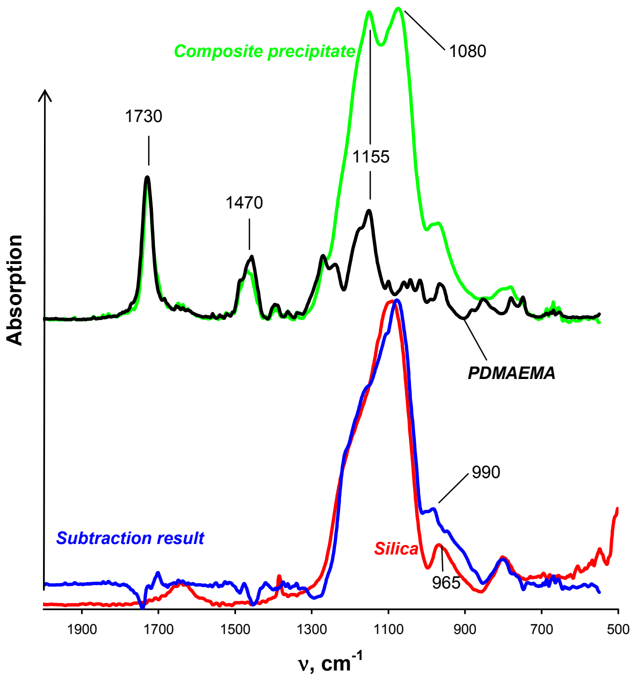

Figure S24. FTIR spectra of PDMAEMA (black line -), pure silica (red line -), PDMAEMA–silica composite (green line -) and the result of subtraction of the PDMAEMA spectrum from the composite spectrum (blue line -): initial SS:DMAEMA = 2.5:1 (25 mM SS:10 mM DMAEMA) and pH 8.5. Pure silica was obtained by precipitation from 50 mM sodium silicate with 1 M HCl at pH 7.0.

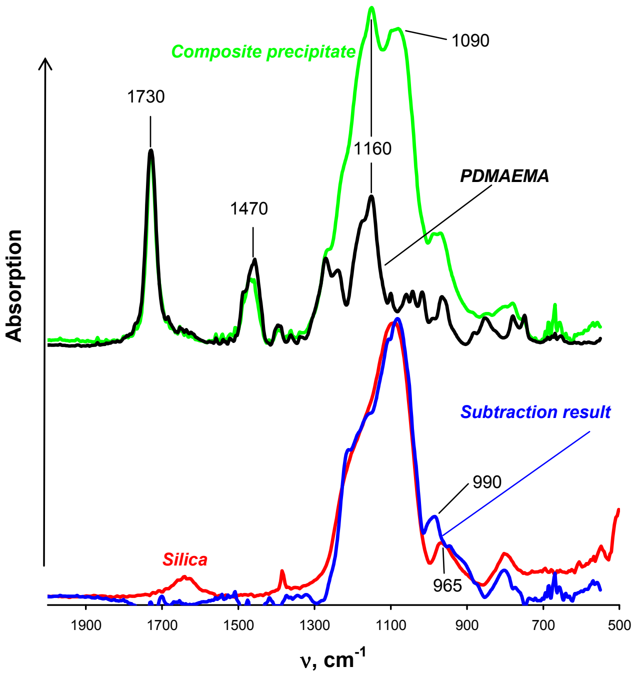

Figure S25. FTIR spectra of PDMAEMA (black line -), pure silica (red line -), PDMAEMA–silica composite (green line -) and the result of subtraction of the PDMAEMA spectrum from the composite spectrum (blue line -): initial SS:DMAEMA = 1:1 (10 mM SS:10 mM DMAEMA) and pH 9.0. Pure silica was obtained by precipitation from 50 mM sodium silicate with 1 M HCl at pH 7.0.

© 2016 by the authors; licensee MDPI, Basel, Switzerland. This article is an open access article distributed under the terms and conditions of the Creative Commons by Attribution (CC-BY) license (http://creativecommons.org/licenses/by/4.0/).
